# Motivational interviewing increases physical activity for adults with low back pain: a systematic review and meta-analysis

**DOI:** 10.1093/abm/kaag040

**Published:** 2026-09-02

**Authors:** Stephen Barrett, Ashley R Dunford, Tara Bewley, Liz Riddell, Gabrielle Barrett, Kane Rodda, Paul O’Halloran, Jeff Breckon, Sarah Hardcastle, Michael Kingsley

**Affiliations:** Research and Innovation, Bendigo Health, Bendigo, VIC 3552, Australia; Holsworth Biomedical Research Centre, La Trobe University, Bendigo, VIC 3552, Australia; Renal Services, Bendigo Health, Bendigo, VIC 3552, Australia; Renal Services, Bendigo Health, Bendigo, VIC 3552, Australia; Physiotherapy & Exercise Physiology Departments, Bendigo Health, Bendigo, VIC 3552, Australia; Loddon Mallee Public Health Unit, Bendigo Health, Bendigo, VIC 3552, Australia; Outpatient Rehabilitation Department, Bendigo Health, Bendigo, VIC 3552, Australia; Centre for Sport and Social Impact, La Trobe University, Melbourne, VIC 3086, Australia; School of Psychology and Public Health, La Trobe University, Melbourne, VIC 3086, Australia; School of Health & Life Sciences, Teesside University, Middlesbrough, TS1 3BA, North Yorkshire, United Kingdom; School of Sport and Physical Activity, Sheffield Hallam University, Sheffield, S1 1WB, United Kingdom; Holsworth Biomedical Research Centre, La Trobe University, Bendigo, VIC 3552, Australia; Department of Exercise Sciences, University of Auckland, Auckland 1023, New Zealand

**Keywords:** low back pain, exercise, motivation, behavior change

## Abstract

**Background:**

Low back pain (LBP) is the leading cause of disability globally and contributes substantially to healthcare costs and lost productivity. Physical activity (PA) is recommended for managing LBP, nevertheless, many individuals with LBP remain insufficiently physically active, and strategies to support behavior change are needed. Motivational interviewing (MI) may support behavior change by enhancing intrinsic motivation and self-efficacy.

**Purpose:**

To synthesize evidence on the effects of MI interventions targeting PA behavior in adults with LBP.

**Methods:**

Six databases (MEDLINE, Embase, CINAHL, Web of Science, CENTRAL, PsycINFO) and clinical trial registries were searched from inception to February 2026. Eligible trials evaluated MI-based interventions targeting PA in adults with acute, subacute, or chronic LBP. Random-effects meta-analyses were conducted to calculate standardized mean differences (SMD; Hedges’ *g*) and 95% confidence intervals (CI). The primary outcome was change in PA; secondary outcomes included pain and disability-related outcomes. Risk of bias was assessed using the Cochrane tool, and certainty of evidence was evaluated using GRADE.

**Results:**

Motivational interviewing-based interventions were associated with a statistically significant increase in PA compared with control conditions (SMD: 0.57; 95% CI, 0.10-1.03; *P* = .02), representing a moderate effect. No statistically significant effects were observed for pain (SMD: 0.04; 95% CI, −0.27 to 0.36; *P* = .76) or disability (SMD: 0.08; 95% CI, −0.25 to 0.40; *P* = .60). Certainty of evidence was low for PA and very low for pain and disability outcomes.

**Conclusions:**

Motivational interviewing interventions were associated with increased PA in adults with LBP but did not demonstrate significant effects on pain or disability. MI may serve as a client-centered support strategy within broader rehabilitation models. Further high-quality trials with fidelity assessment and longer follow-up are warranted.

## Introduction

Low back pain (LBP) is the leading cause of years lived with disability worldwide, affecting more than 570 million people at any one time and contributing substantially to healthcare costs and lost productivity.^1^^,^^2^ Most of the societal burden arises from individuals experiencing chronic or recurrent symptoms, for whom recovery is often slow, incomplete, and associated with persistent functional limitations.^3–5^ Despite significant research investment, uncertainty remains about how best to support individuals with ongoing LBP in maintaining health and function over the long term.^5^

Physical activity (PA) is a key target in managing LBP, particularly chronic presentations, where consistent movement is associated with improved function, reduced recurrence risk, and broader health benefits.^6^ A recent critical narrative review found that a range of approaches, including general exercise and multidisciplinary interventions can lower pain and work disability in chronic LBP.^7^ Evidence for PA in acute (under 6 weeks) and subacute (6 to 12 weeks) LBP remains limited, and the optimal type, intensity, and mode of delivery remain unclear. Despite the benefits, PA engagement remains low among people with LBP, with many not meeting recommended levels of at least 150 minutes of moderate-intensity aerobic activity per week plus 2 sessions of strength-based exercise.^6^ Barriers to activity are numerous, and participants often report a combination of low confidence, inconsistent messaging from health professionals, uncertainty about appropriate activity, limited knowledge regarding safe movement, fear of symptom exacerbation, and difficulties adopting and sustaining self-directed behavior change.^8–10^ Addressing these challenges requires behavior change approaches that are flexible, patient-centered, and responsive to individual needs.

Motivational interviewing (MI) is a collaborative counseling approach that aims to strengthen an individual’s intrinsic motivation and readiness for change.^11^ A typical MI session involves a structured yet flexible client-centered conversation about change guided by open-ended questions, affirmations, reflective listening and summaries, with the clinician seeking to elicit “change talk” and support autonomy through the processes of engaging, focusing, evoking, and planning.^11–13^ It has demonstrated effectiveness across a range of health behaviors, including PA,^13–16^ smoking cessation,^17^^,^^18^ and dietary change.^19^ A previous review of MI in people with chronic LBP evaluated its use when combined with structured exercise, focusing primarily on symptom outcomes such as pain intensity.^20^ That review found that MI in addition to exercise did not result in significant changes in pain, disability or physical function, potentially because the primary mechanism of MI (supporting self-directed behavior change) was not aligned with the outcomes assessed.^20^ More broadly, meta-analyses examining MI across chronic health conditions have reported small but positive effects on PA,^15^ while reviews in musculoskeletal pain populations have focused primarily on symptom-related outcomes rather than behavioral change.^20^^,^^21^ These findings highlight both the potential and the uncertainty surrounding MI in LBP contexts.

To our knowledge, no prior review has specifically synthesized evidence on MI interventions targeting PA behavior in adults with LBP. This review addresses that gap by focusing specifically on studies that used MI interventions to increase PA, regardless of whether it was delivered as a stand-alone strategy or part of a broader intervention. Variability in delivery mode, provider discipline, and comparator type may also influence intervention effectiveness and warrant examination. By selecting studies where PA was the primary behavioral target, we aimed to directly assess the effectiveness of MI when used as a behavior change tool. While MI originated as a counseling approach centered on practitioner empathy and client autonomy, more recent theoretical work has aligned MI with self-determination theory (SDT), which posits that fostering intrinsic motivation through autonomy-supportive interactions can support sustained behavior change.^22^ This theoretical framing strengthens the rationale for using MI in the context of LBP, where ambivalence about PA engagement and fear-avoidance behaviors are common.

Although MI primarily targets cognitive and behavioral processes rather than symptom mechanisms directly, PA is a core component of guideline-based management for LBP.^23^ If MI successfully increases PA, it is clinically important to examine whether such behavioral change translates into improvements in pain and disability outcomes. Evaluating this potential pathway clarifies whether behavior change alone is sufficient to influence symptom-related outcomes or whether integration with pain-specific ­strategies is required.

Therefore, the research questions for this systematic review were:

1. What is the effect of MI interventions on PA engagement in adults with LBP?
2. What are the effects of MI interventions on pain and disability in this population?

## Methods

This systematic review and meta-analysis were conducted and reported according to the Preferred Reporting Items for Systematic Reviews and Meta-Analyses (PRISMA) (Supplementary Material S1).^24^ This review was prospectively registered with PROSPERO (registration ID: CRD42024585036).

### Search strategy

Eligible studies were identified from database inception to June 2025 via systematic searches in MEDLINE, Embase, CINAHL, Web of Science, PsycINFO and The Cochrane Central Register of Controlled Trials (CENTRAL) electronic databases. Database-specific search strategies were developed with the guidance of professional clinical librarians. Search terms included words relating to MI, LBP, PA, pain, and disability (see Supplementary Material S2 for the search strategy). Manual searches of relevant systematic reviews and reference lists were conducted. Although the PROSPERO protocol listed PubMed, MEDLINE was searched directly as planned. This reflects a typographical error in the PROSPERO registration rather than a deviation from the intended search strategy. Scopus, which was originally specified in the protocol, was not searched due to overlap with other databases. This represents a minor deviation from the protocol.

### Study selection criteria

We included randomized controlled trials involving adults aged 18 years or older with acute LBP (under 6 weeks), subacute LBP (6 to 12 weeks), or chronic LBP (more than 12 weeks). Eligible studies evaluated interventions that used MI as a central strategy to promote PA. Motivational interviewing could be delivered in person, by telephone, or through telehealth, either as a stand-alone intervention or as part of a multicomponent program. Interventions were required to include at least one session delivered in a one-to-one format. Control groups included usual care, standard PA advice, or other behavior change interventions not based on MI.

Studies were required to report PA as a primary or secondary outcome, measured using either objective methods (such as accelerometry or pedometers) or subjective methods (such as self-report questionnaires). Secondary outcomes included pain and disability; these secondary outcomes were included to evaluate whether behavioral changes in PA translated into clinically meaningful endpoints in LBP populations. Studies published as conference abstracts only, pilot or feasibility trials without sufficient outcome data, and studies not published in English were excluded.

### Screening and data extraction

Five reviewers (S.B., A.D., T.B., L.R., G.B.) screened the titles and abstracts using Covidence (Covidence Systematic Review Software, Veritas Health Innovation, Melbourne, Australia). Each study was independently assessed by 2 reviewers against pre-specified eligibility criteria. Discrepancies at the title and abstract stage were resolved through discussion; where consensus was not achieved, a third reviewer adjudicated.

Full-text articles of potentially relevant studies were retrieved and independently assessed against pre-specified eligibility criteria by 2 reviewers. Disagreements were resolved through discussion or consultation with a third reviewer. Reasons for exclusion at the full-text stage were recorded and are reported in the PRISMA flow diagram.

Two reviewers (S.B. and one of A.D., T.B., L.R., G.B.) independently extracted data using a structured extraction form developed by the review team and pilot-tested prior to use to ensure consistency between reviewers. Extracted data included study design, setting, sample size, participant characteristics, intervention details (including delivery format, frequency, duration, provider background, and fidelity strategies), comparator characteristics, and outcome measures (definitions, measurement tools, time points).

Fidelity strategies were defined as evidence-based procedures used to monitor or enhance adherence to MI principles and delivery quality to increase the probability that the intervention was delivered as intended. Examples included formal training programs, structured supervision or feedback processes, audio-recording review, and use of validated coding tools such as the Motivational Interviewing Treatment Integrity (MITI) scale.^25^ The MITI assesses both technical components (eg, elicitation of change talk) and relational components (eg, empathy and partnership) of MI delivery. Fidelity data were extracted descriptively from included studies where such data were reported.

For continuous outcomes, we extracted post-intervention means and standard deviations. Where standard deviations were not reported, they were estimated from alternative measures of dispersion (eg, standard errors, confidence intervals, or *P-*value) following procedures outlined in the Cochrane Handbook for Systematic Reviews of Interventions.^26^ When data were reported as medians with interquartile ranges, means and standard ­deviations were approximated using the method proposed by Hozo et al.^27^

### Risk of bias

The risk of bias for each included study was independently assessed by 3 reviewers (S.B. and one of A.D., T.B., G.B.) using the Cochrane Risk of Bias tool.^28^ Each domain was rated as having a “low,” “high,” or “unclear” risk of bias, with disagreements resolved through discussion or consultation with a third reviewer, if necessary. For sensitivity analyses, studies were classified as overall high risk of bias if one or more domains were rated as high risk. Detailed domain-level risk-of-bias assessments for each study are provided in Supplementary Material S3.

### Certainty of evidence

To evaluate the overall certainty of evidence across outcomes, the Grading of Recommendations, Assessment, Development and Evaluation (GRADE) framework was applied using GRADEpro Guideline Development Tool^29^ (GRADEpro GDT, McMaster University, United States, version 3.6). The quality of evidence for each meta-analyzed outcome was initially rated as high and downgraded based on predefined criteria, including risk of bias, inconsistency, indirectness, imprecision, and potential for publication bias. Downgrading decisions were made independently by 2 reviewers and resolved through consensus where required. Certainty ratings are reported using standard GRADE terminology to reflect confidence in the body of evidence, consistent with GRADE guidance on formulating informative statements that combine effect size and certainty.^30^

### Statistical analysis

All analyses were conducted using end-point data, with means and standard deviations extracted from each study’s post-intervention time point. Standardized mean differences were calculated using Hedges’ *g* (a small-sample corrected standardized mean difference), as implemented in Review Manager (RevMan), to account for varied measurement tools across studies.

Random-effects models were used to account for anticipated clinical and methodological heterogeneity across interventions, LBP duration (acute, subacute, chronic), and delivery contexts. Statistical heterogeneity was evaluated using the chi-squared (χ^2^) test and quantified using the *I*^2^ statistic, with values of 25%, 50%, and 75% representing low, moderate, and high heterogeneity, respectively. The magnitude of pooled Hedges’ *g* values was interpreted using conventional thresholds for standardized mean differences (0.2 small, 0.5 moderate, 0.8 large).^31^

Pre-specified subgroup analyses were conducted to explore potential sources of heterogeneity. Subgroup analyses were performed using stratified random-effects models within RevMan. Statistical tests for subgroup differences were used to examine whether pooled effects differed between categories. These analyses were exploratory and were not conducted as formal meta-regression tests of moderation.

Subgroup analyses were conducted based on pre-specified characteristics: LBP duration (acute/subacute vs chronic), intervention delivery mode (face-to-face vs remote), comparator type (usual care vs active treatment), intervention dose (≤3 sessions vs ≥4 sessions), and professional delivering the intervention (physiotherapist vs other health professional). One additional subgroup was conducted for PA outcomes measured using objective devices only.

Sensitivity analyses were conducted by excluding studies rated as having a high risk of bias to assess the robustness of pooled estimates. Publication bias was assessed for the primary outcome through visual inspection of funnel plots. Formal statistical tests for funnel plot asymmetry (eg, Egger’s regression) were not performed due to the small number of included studies (<10), consistent with methodological guidance.^26^ All statistical analyses were conducted using RevMan.

Planned analysis by intervention duration (short-term vs long-term) was not conducted due to poor reporting in the included studies. Two additional outcomes (quality of life and adherence to PA recommendations) were specified in the protocol but were reported too infrequently and inconsistently to allow meaningful synthesis. This represents a deviation from the protocol, justified by insufficient available data.

## Results

### Study selection

**Figure 1:**
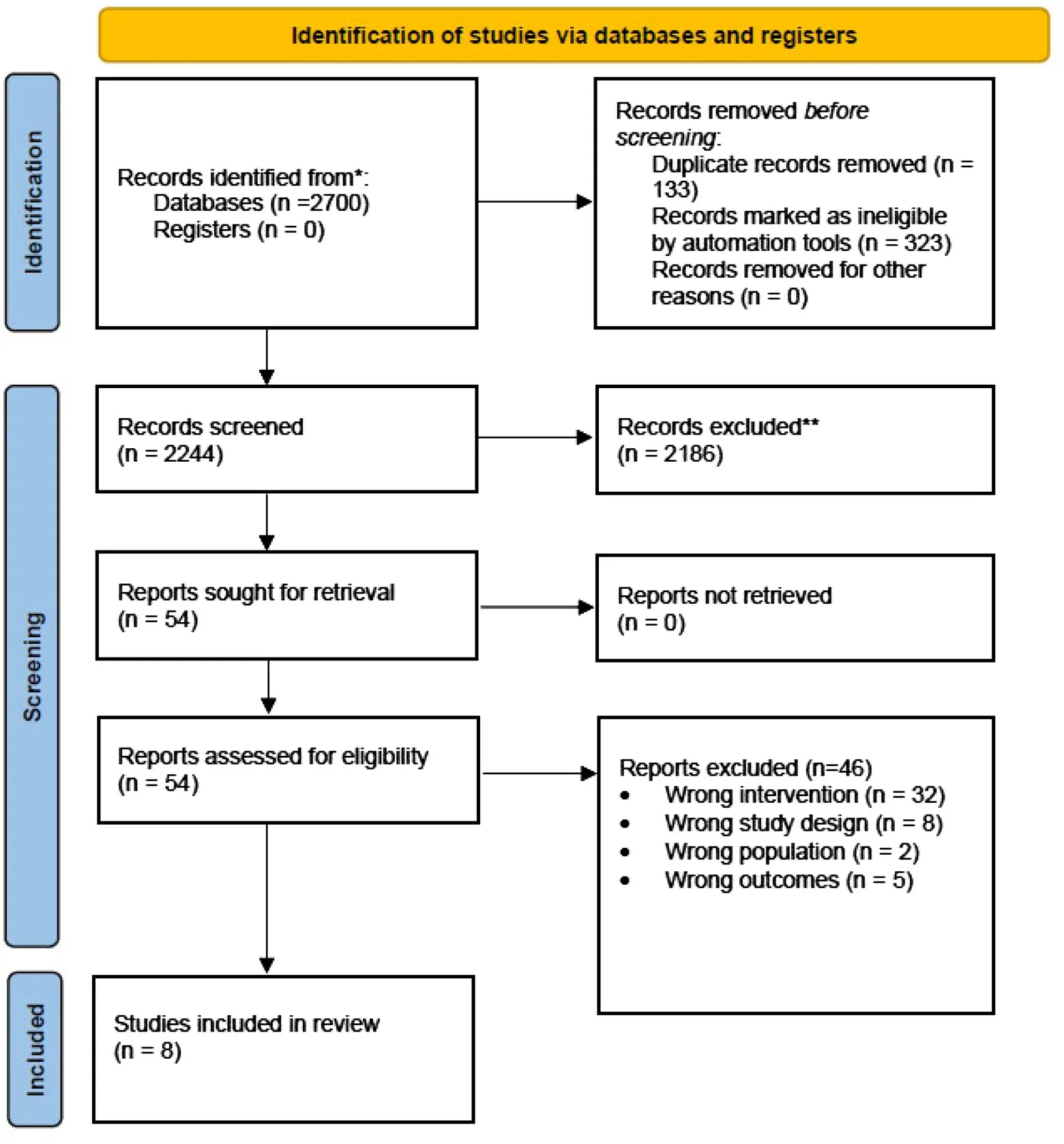
PRISMA flow diagram.

Following de-duplication, 2244 studies were screened. The PRISMA diagram illustrating the screening process is shown in Figure 1. Eight full-text articles fulfilled the inclusion criteria and were included in the quantitative synthesis.^32–39^ (Table 1). See Supplementary Material S4 for a summary of the excluded papers.

**Table 1:** Characteristics of included studies.

Study	*N* (% female)	Mean age (SD)	Population & Setting	Delivery mode	MI components	Dose (sessions × duration)	Provider (training)	Fidelity	Comparator	Outcomes
**Amorim, 2019^32^**	68 (50)	58 (13)	Chronic LBP; outpatient physiotherapy & community	Mixed (F2F + phone + app)	MI coaching + goal setting + app support	1 F2F + 12 phone over 6 months	Health coach (Wellness Coaching Australia)	Not stated	Phone goal advice	PA (Accelerometer); Pain (NRS); Disability (RMDQ)
**Ben-Ami, 2017^33^**	220 (54)	42 (8)	Chronic LBP; outpatient physiotherapy	Face-to-face	TTM-based MI	6 weekly	Physical therapists (2-day training + supervision)	Not assessed	Standard PT	PA (BPAQ); Pain (NRS); Disability (RMDQ)
**Holden, 2024^34^**	46 (52)	44 (14)	Subacute LBP; outpatient physiotherapy	Mixed (F2F + app)	MI consult + MiMate app	6 weekly 30 min	Physical therapists	MITI 4.2.1	Standard PT	PA (Accelerometer); Pain (PSEQ); Disability (ODI)
**Iles, 2011^35^**	30 (40)	40 (12)	Non-chronic LBP; outpatient physiotherapy	Telephone	MI coaching	5 over 7 weeks	Physiotherapists	Not stated	Standard PT	PA (PSFS); Pain (PSEQ); Disability (ODI)
**Leonhardt, 2008^36^**	1378 (68)	48 (14)	LBP in primary care; general practice	Face-to-face	Motivational counselling	3 sessions	Practice nurses	Not stated	Standard care	PA (FQPA); Pain (VK); Disability (HFAQ)
**Rodrigues, 2021^39^**	60 (50)	NS	Adults with LBP; hospital physiotherapy	Face-to-face	Single-session MI	1 session	Physiotherapists	Not stated	Standard PT	PA (Adherence); Pain (VPIS); Disability (RMDQ)
**Shimo, 2021^37^**	37 (0)	45 (12)	LBP; workplace setting	Face-to-face	PA education + MI counselling	1 education + 12 weekly	PT + occupational nurses	Not stated	Education only	PA (Accelerometer); Pain (VAS); Disability (RMDQ)
**Vong, 2011^38^**	76 (63)	45 (11)	Chronic LBP; outpatient physiotherapy	Face-to-face	MI-based counselling	10 weekly 30 min	Physiotherapists	Not stated	Standard PT	PA (Compliance); Pain (NRS); Disability (RMDQ)

Abbreviations: BPAQ, Baecke Physical Activity Questionnaire; F2F, face-to-face; FQPA, Freiburg Questionnaire for Physical Activity; HFAQ, Hannover Functional Ability Questionnaire; MI, motivational interviewing; MITI, Motivational Interviewing Treatment Integrity; NRS, Numerical Rating Scale; NS, not stated; ODI, Oswestry Disability Index; PA, physical activity; PSEQ, Pain Self-Efficacy Questionnaire; PT, physiotherapy/physical therapy; RMDQ, Roland-Morris Disability Questionnaire; TTM, Transtheoretical Model; VAS, Visual Analogue Scale; VK, Verbal Category Scale; VPIS, Verbal Pain Intensity Scale.

The included studies were conducted across 6 countries, with the largest representation from Australia (*n* =  3),^32^^,^^34^^,^^35^ followed by individual studies from Israel,^33^ Germany,^36^ Japan,^37^ Portugal,^39^ and Canada^38^ (*n* =  1 each). Participants were primarily adults with LBP recruited from outpatient physiotherapy departments in hospital settings,^32^^,^^34^^,^^35^^,^^38^^,^^39^ with 1 study that included individuals recruited from the general community^33^ and 1 involving a workplace population comprising full-time Japanese office workers and mechanics.^37^

### Study characteristics

All included studies involved behavioral interventions incorporating MI with the aim of increasing PA in individuals with LBP. Interventions were delivered across a variety of formats, including face-to-face, telephone, and app-based modalities. Common features included individualized delivery, flexible session structures, and integration into physiotherapy consultations.

Several studies employed digital supports such as smartphone apps or activity trackers to reinforce behavior change outside of formal sessions.^32^^,^^34^ For example, 1 study combined MI with a mobile app (MiMate) featuring interactive modules, diaries, and a flare-up support tool alongside physiotherapy.^34^ Amorim et al.^32^ used a web-based platform (IMPACT app) alongside coaching calls and weekly motivational messages to reinforce PA goals.

The MI techniques described across studies focused on increasing intrinsic motivation, building autonomy, and supporting self-efficacy to promote behavior change. Common components included goal setting,^32–34^ eliciting change talk through structured questions or app modules,^32^^,^^34^ and stage-matched counseling based on the transtheoretical model.^33^ Other studies emphasized enhancing PA self-efficacy and behavioral readiness.^33^^,^^38^^,^^39^ Notably, few studies explicitly described the relational components of MI, such as the MI spirit (comprising collaboration, acceptance, compassion and evocation). While OARS (open questions, affirmations, reflective listening, and summaries) were mentioned, the omission of relational strategies may represent a limitation in both intervention delivery and reporting.

Where reported, training in MI ranged from structured short courses^32^^,^^34^ to multi-day workshops with supervision.^33^^,^^37^ Training content typically covered MI principles, communication techniques, behavior change theory (eg, transtheoretical model), and skills for embedding MI within physiotherapy practice. Not all studies reported the training received by intervention providers.^38^

Fidelity of MI delivery was variably reported, with most studies providing limited or no detail on fidelity monitoring procedures. Only 1 study undertook a comprehensive fidelity assessment, using both provider-rated (MITI 4.2.1) and patient-reported (Client Evaluation of Motivational Interviewing, CEMI) tools to evaluate adherence to MI principles.^34^ One additional study recorded fidelity through documentation of patients’ stage of change at baseline and discharge,^33^ although this provides only indirect evidence of MI delivery quality. The remaining studies either did not report fidelity procedures or did not indicate that fidelity assessments were conducted.

### Risk of bias

**Figure 2:**
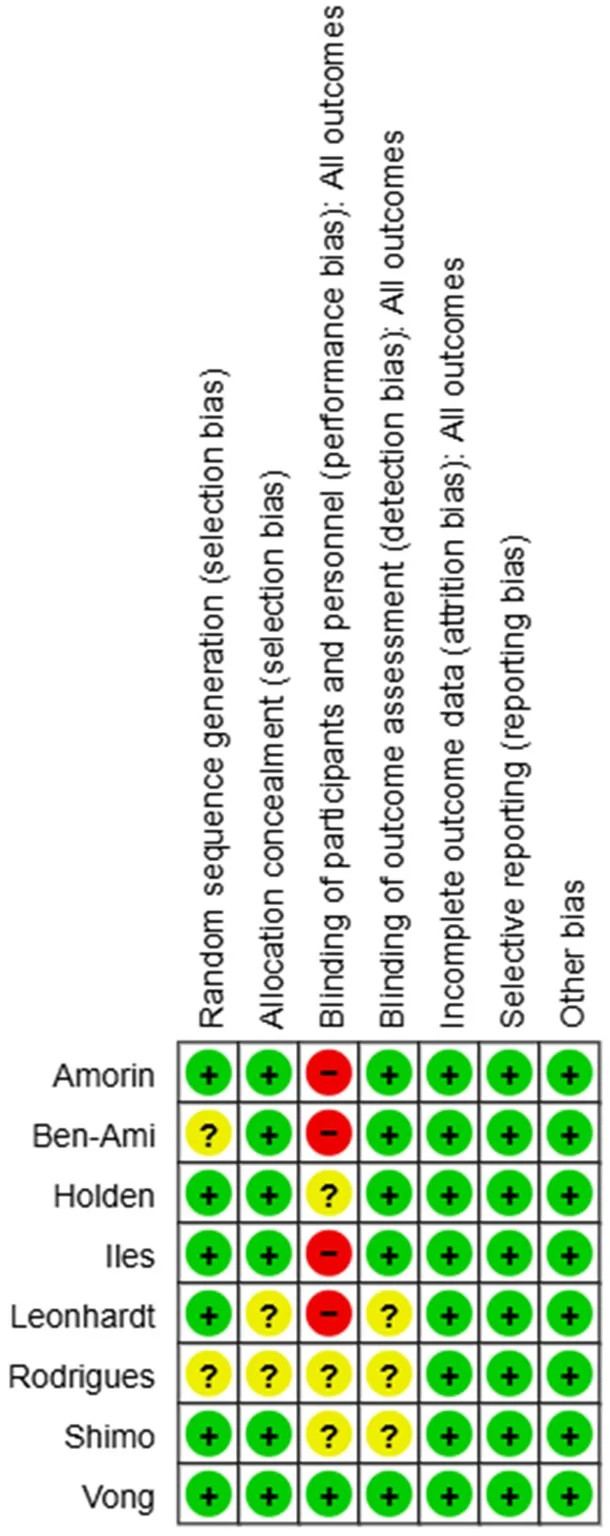
Risk of bias of included studies.

The risk of bias assessment for each included study is summarized in Figure 2. As is common in trials evaluating behavior change interventions, blinding of participants and personnel was difficult. Consequently, 4 studies (50%) were judged to have a high risk of bias due to the lack of blinding. Three studies did not provide sufficient detail on blinding of outcome assessors and were rated as unclear. All studies demonstrated a low risk of bias for selective reporting, allocation concealment, and other sources of bias.

### GRADE assessment

The overall certainty of evidence for the effectiveness of MI interventions for changes in PA, pain and disability-related outcomes is presented in Table 2.

**Table 2:** Summary of findings table.

Outcome	**Anticipated absolute effects** ^a^ **(95% CI)**	No of participants (studies)	Certainty of the evidence (GRADE)	Informative statements
**Physical activity**	SMD 0.57 higher [0.10 to 1.03]	1392 (8 RCTs)	⨁⨁◯◯ Low^b^^,^^c^^,^^d^^,^^e^	The evidence suggests motivational interviewing results in an increase in overall physical activity for people with low back pain
**Pain**	SMD 0.04 higher [−0.27 to 0.36]	1392 (8 RCTs)	⨁◯◯◯ Very low^b^^,^^c^^,^^d^^,^^e^	The evidence is very uncertain about the effect of motivational interviewing on pain for people with low back pain
**Disability-related outcomes**	SMD 0.08 higher [−0.25 to 0.40]	1392 (8 RCTs)	⨁◯◯◯ Very low^b^^,^^c^^,^^d^^,^^e^	The evidence is very uncertain about the effect of motivational interviewing on disability-related outcomes for people with low back pain

Abbreviations: RCT, randomized controlled trial; SMD, standardized mean difference.

a The risk in the intervention group (and its 95% confidence interval) is based on the assumed risk in the comparison group and the relative effect of the intervention (and its 95% CI).

b Low number of studies with high risk of bias.

c High heterogeneity.

d Differences in population and outcome measures.

e Wide confidence intervals.

### Effects of MI interventions on changes in PA

**Figure 3:**
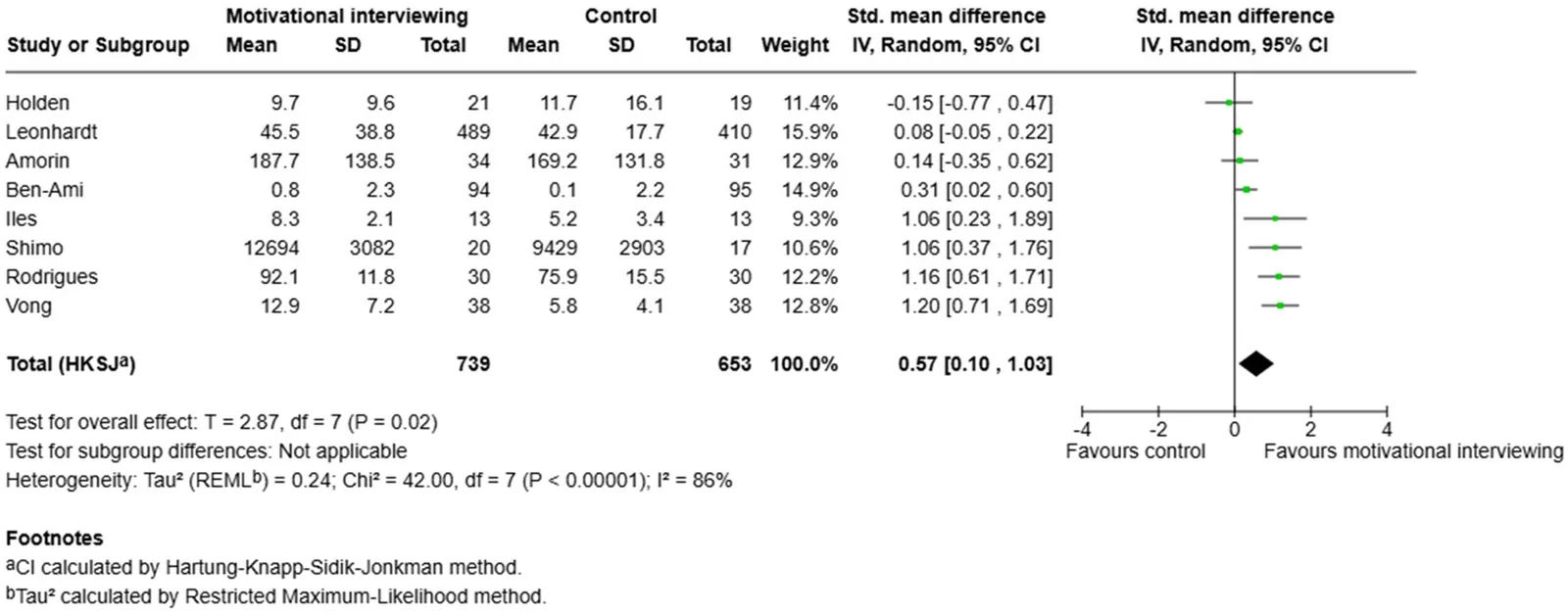
Meta-analysis investigating motivational interviewing interventions for changes physical activity.

All 8 included studies provided data on changes in PA for the intervention and control groups at the post-intervention follow-up and were included in the meta-analysis. The meta-analysis for MI versus standard care for change in PA demonstrated a significant effect in favor of MI (SMD: 0.57; 95% CI, 0.10-1.03, *P* = .02; Figure 3). Statistical heterogeneity was substantial (*I*^2^ = 86%), indicating ­variability in effect estimates across studies. Visual inspection of the funnel plot for PA did not suggest marked asymmetry (Supplementary Materials S13-S15); however, interpretation is limited by the small number of included studies (*n* = 8). Overall, the evidence suggests MI results in an increase in overall PA for people with LBP.

### Effects of MI interventions on changes in pain

**Figure 4:**
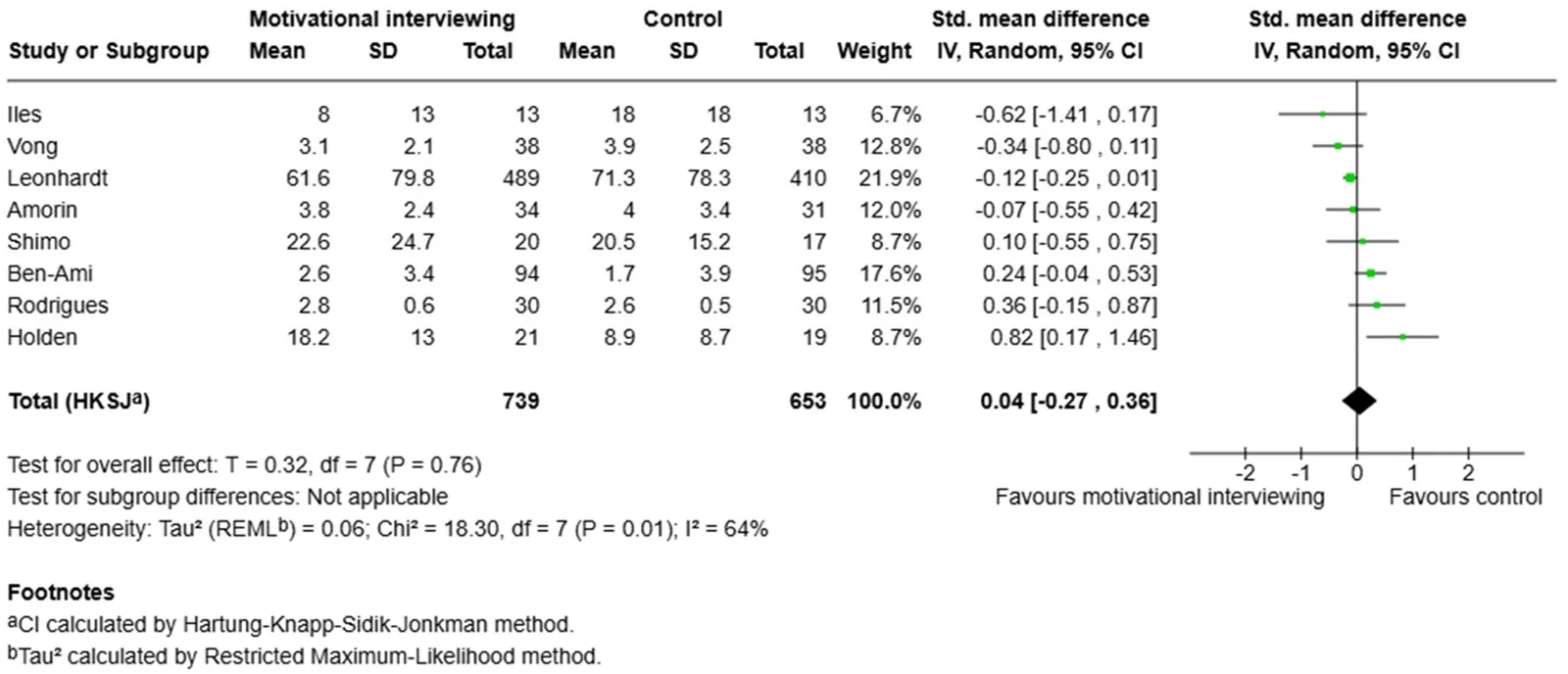
Meta-analysis investigating motivational interviewing interventions for changes pain.

All 8 included studies provided data on changes in pain and were included in the meta-analysis. Motivational interviewing was not associated with a statistically significant effect on pain (SMD: 0.04; 95% CI, −0.27 to 0.36, *P* = .76; Figure 4). Statistical heterogeneity was moderate to substantial (*I*^2^ = 64%). Overall, the evidence is very uncertain about the effect of MI on pain for people with LBP.

### Effects of MI interventions on changes in disability-related outcomes

**Figure 5:**
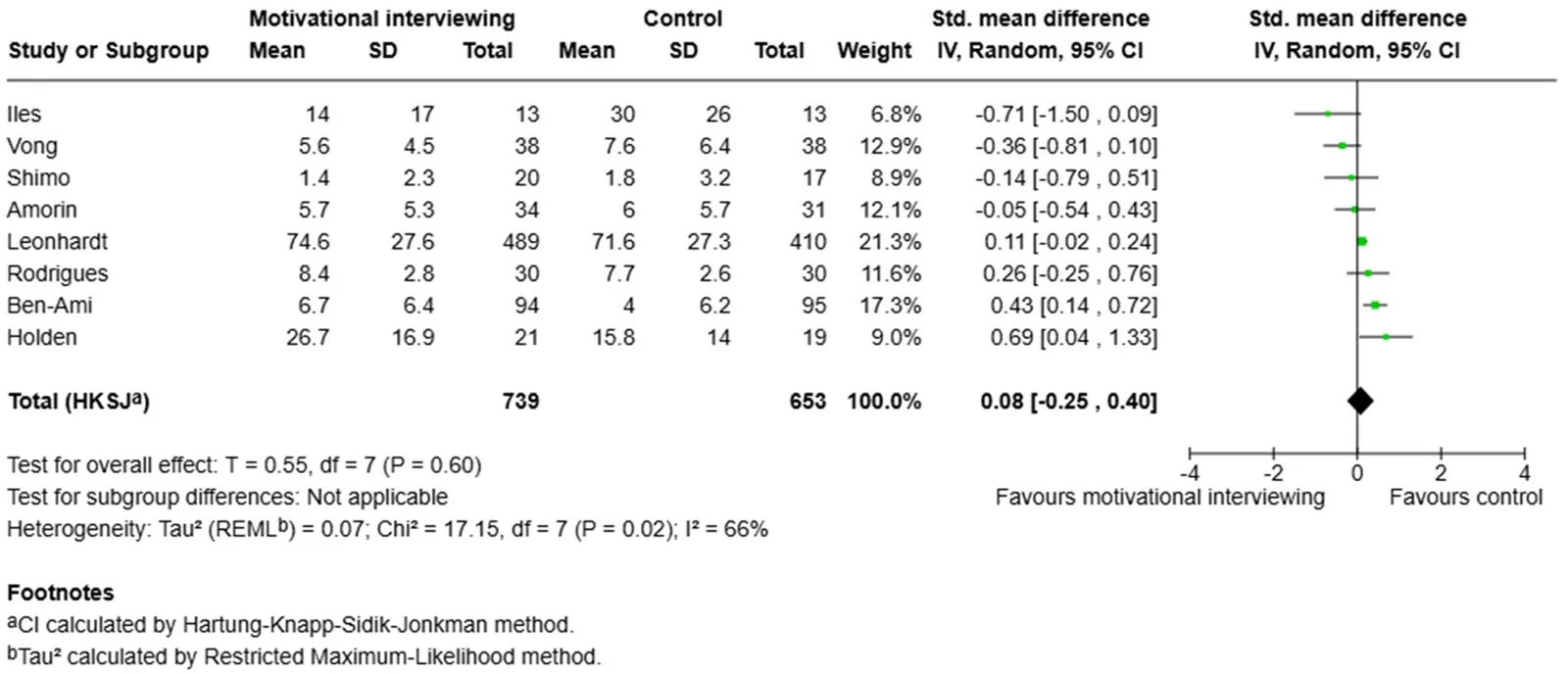
Meta-analysis investigating motivational interviewing interventions for changes disability-related outcomes.

All 8 included studies provided data on changes in disability-related outcomes and were included in the meta-analysis. MI was not associated with a statistically significant effect on disability (SMD: 0.08; 95% CI, −0.25 to 0.40, *P* = .60; Figure 5). Statistical heterogeneity was moderate to substantial (*I*^2^ = 66%). Overall, the evidence is very uncertain about the effect of MI on disability-related outcomes for people with LBP.

### Subgroup and sensitivity analyses

Pre-specified subgroup analyses for PA outcomes are summarized in Table 3. Forest plots for subgroup and sensitivity ­analyses are presented in Supplementary Materials S5-S12. Comparator type and provider discipline demonstrated statistically significant subgroup differences. MI showed a significant effect when ­compared with active treatment and when delivered by physiotherapists. Tests for subgroup differences were significant for comparator type (χ^2^ = 8.24, *P* = .004) and provider discipline (χ^2^ = 8.24, *P* = .004).

**Table 3:** Subgroup analyses for physical activity (random-effects model).

Characteristics	No. of studies	No. of participants (intervention/control)	SMD (95% CI)	*P* (within subgroup)	*I* ^2^ (%)	*P* (for subgroup difference)
**Full analysis**	8	1392 (739/653)	0.57 [0.10, 1.03]	**.02**	86	-
**Delivery mode**						.94
**Face-to-face**	6	1301 (692/609)	0.58 [−0.04, 1.21]	.06	90	
**Phone**	2	91 (47/44)	0.54 [−5.30, 6.37]	.45	72	
**Pain duration**						**.004**
**Chronic pain**	5	427 (216/211)	0.74 [0.10, 1.38]	**.03**	78	
**Acute/Sub-acute pain**	3	965 (523/442)	0.25 [−1.18, 1.68]	.53	75	
**Comparator type**						.97
**Versus active treatment**	6	428 (216/212)	0.75 [0.16, 1.34]	**.02**	77	
**Versus no intervention**	2	964 (523/441)	0.09 [−0.08, 0.25]	.09	0	
**Sessions**						.97
**≤3 sessions**	2	959 (519/440)	0.59 [−6.20, 7.38]	.47	93	
**≥4 sessions**	6	433 (220/213)	0.57 [−0.02, 1.17]	.06	76	
**Provider discipline**						**.004**
**Physio delivered**	6	428 (216/212)	0.75 [0.16, 1.34]	**.02**	77	
**Other health professional**	2	964 (523/441)	0.09 [−0.08, 0.25]	.09	0	
**Measurement tool**						.38
**Device measured**	3	142 (75/67)	0.33 [−1.21, 1.86]	.46	74	
**Self-reported**	5	1250 (664/586)	0.70 [0.03, 1.37]	**.04**	89	

No statistically significant subgroup differences were observed for delivery mode, pain duration, session number, or PA measurement method (device vs self-report). Subgroup analyses were exploratory and based on small numbers of studies per category; findings should therefore be interpreted cautiously.

Sensitivity analyses excluding studies classified as high risk of bias (excluding performance bias alone) yielded a larger but nonsignificant pooled estimate for PA (SMD 0.83; 95% CI, −0.20 to 1.85). Effects for pain (SMD 0.20; 95% CI, −0.57 to 0.95) and disability (SMD 0.09; 95% CI, −0.64 to 0.81) remained nonsignificant. The direction of effect was consistent with the primary analysis, although precision was reduced due to the smaller number of included studies.

## Discussion

The results of this meta-analysis of 8 randomized controlled trials indicate that MI was associated with a moderate positive effect on PA in individuals with LBP. These positive findings are noteworthy, as even modest improvements in PA can lead to meaningful health benefits and improved musculoskeletal function.^40^ No significant effects were observed for pain or disability-related outcomes. The certainty of the evidence was rated as low for PA outcomes and very low for changes in pain and disability-related outcomes.

The pooled effect size for PA (SMD = 0.57) exceeds that reported in previous reviews incorporating MI to improve PA.^15^^,^^20^^,^^41^^,^^42^ O’Halloran et al.^15^ identified a smaller effect (SMD = 0.19) for MI across chronic health conditions, and a recent review found no effect of MI combined with exercise on physical function in people with chronic LBP (SMD = 0.00).^20^ The larger effect observed in our review may reflect our focus on studies in which PA was the primary behavioral target, rather than a secondary or intermediate outcome. It is also possible that adults with LBP represent a population particularly responsive to MI-based PA interventions. However, given the small number of included trials and substantial heterogeneity, it is not possible to determine whether the observed effect size reflects population-specific responsiveness or methodological differences across reviews. This warrants further investigation in larger, well-powered trials.

Including studies across the spectrum of LBP (acute to chronic) may also have contributed to variability in effect estimates. Subgroup analyses in our review showed similar, though not statistically significant, effects in both chronic (SMD = 0.64) and acute/subacute (SMD = 0.50) LBP groups, suggesting that MI may have relevance for improving PA across the spectrum of LBP presentations. However, statistical heterogeneity was substantial, and pooled estimates should therefore be interpreted cautiously. The consistent direction and relative magnitude of effect across subgroups support the potential clinical relevance of MI for increasing PA in this population.

A review of behavioral interventions incorporating MI reported an SMD of 0.45 for increases in total PA, corresponding to approximately 1323 additional steps per day.^41^ Applying a similar approximation, the SMD of 0.57 in this review may correspond to approximately 1675 additional steps/day. Marshall et al.^43^ suggest that an increase of 1000 steps/day equates to about 10 minutes of moderate intensity activity, indicating that MI interventions could add approximately 17 minutes of moderate activity to daily routines. These extrapolations are illustrative only and should not be interpreted as direct estimates of behavioral change in this population. Step count increases, especially in LBP populations may reflect light-intensity activity rather than moderate-to-vigorous PA. Only a small number of studies used objective monitoring devices, and in the subgroup analysis limited to these studies, the direction of effect remained positive but was not statistically significant. Nevertheless, even small increments in daily PA are associated with reductions in all-cause mortality, cardiometabolic risk, and improved musculoskeletal health.^44^^,^^45^ In the context of LBP, regular PA supports physical function, reduces recurrence risk, and facilitates long-term self-management.^7^^,^^46^

Motivational interviewing may therefore serve as a pragmatic approach to initiating movement behavior change in populations where structured exercise may be inaccessible or overwhelming. Although traditional MI requires specific training and is commonly delivered one-to-one, several of the included studies utilized brief formats, telephone delivery, or digitally supported approaches, suggesting potential flexibility in implementation. As such, MI may be best conceptualized as a flexible adjunct within rehabilitation models rather than as a stand-alone intervention.

Despite improvements in PA, MI did not lead to significant changes in pain or disability-related outcomes. The findings are consistent with prior reviews^20^^,^^21^ and may reflect the nature of the included interventions. Most were designed specifically to increase PA, rather than to directly address pain mechanisms or functional limitations. Increases in PA do not necessarily translate into reductions in pain in LBP populations, particularly when interventions do not explicitly target pain-related mechanisms.^47^^,^^48^ Pain in the context of LBP is a complex, biopsychosocial experience shaped by prior injury, central sensitization, emotional distress, maladaptive beliefs, and behavioral responses such as fear avoidance or catastrophizing.^49–51^ Interventions such as cognitive behavioral therapy, graded exposure, and pain neuroscience education directly target these dimensions and have demonstrated stronger effects on pain and function in LBP populations.^52^^,^^53^ In contrast, the MI techniques described in the included studies such as goal setting, eliciting change talk, enhancing self-efficacy do not explicitly address maladaptive pain beliefs or nervous system sensitization. PA behavior change alone may therefore be insufficient to modify symptom severity without integration of complementary strategies. Disability-related outcomes may take longer to shift, particularly when interventions are relatively brief or not tailored to functional goals. The absence of effect on these outcomes reinforces the need to align intervention mechanisms with the specific outcome being targeted.

### Strengths and limitations

This review has several strengths. It focused specifically on MI interventions in LBP where PA was an explicit behavioral target, allowing more precise estimation of behavior-specific effects. The review incorporated quantitative synthesis, subgroup analyses, sensitivity analyses, and GRADE assessments, providing a structured appraisal of effect magnitude and certainty. A particular strength of this review is its explicit consideration of treatment fidelity, a dimension rarely synthesized in MI reviews within musculoskeletal populations.

Some limitations should also be considered when interpreting the results. Only 8 studies were eligible for inclusion, limiting the precision of pooled estimates and the ability to detect subgroup effects. Outcome measures and intervention designs varied substantially, contributing to statistical heterogeneity. Fidelity to MI delivery and detail on the specific MI techniques were poorly reported. Only one study used validated fidelity tools,^34^ while the majority did not report fidelity assessment at all. Training of intervention providers was also variably reported and inconsistently described. In some cases, it was unclear whether interventions adhered to core MI principles or merely incorporated selected strategies inspired by MI. For example, scripted app modules may not reflect authentic MI processes, particularly in the absence of relational elements such as evocation, affirmation, and reflective listening.

Beyond technical fidelity, relational techniques that underpin MI spirit or therapeutic alliance are likely to influence behavioral outcomes but were not assessed in the included trials.^53^ Constructs such as collaboration, empathy, and autonomy support are theoretically central to MI and may contribute meaningfully to behavior change, yet were not systematically measured.

Few studies clearly reported the specific MI techniques used—either relational (eg, expressing empathy, supporting autonomy) or content-based (eg, goal setting, importance/confidence rulers). The development of MI taxonomies and tools such as the MITI coding system was intended to support consistent reporting and help identify which combinations of techniques are most effective.^54^ Relational techniques central to MI may also enhance the effectiveness of content-based behavior change techniques more broadly, regardless of theoretical orientation, suggesting that interpersonal style warrants greater attention in future intervention design and reporting.^55^ Without such detail, advancing ­understanding of MI’s active ingredients and replicating effective approaches remains challenging. These limitations restrict interpretability and may underestimate the impact of high-fidelity MI interventions.

The overall certainty of the evidence was rated as low to very low, reflecting study limitations including risk of bias, inconsistency and imprecision. Accordingly, the true effect of MI on PA, pain and disability-related outcomes may differ from the estimates reported. Future studies should incorporate robust fidelity monitoring and clearer reporting of MI-specific components to enhance internal validity and support implementation in clinical practice.

## Conclusion

Motivational interviewing appears to be a promising client-centered strategy for increasing PA in individuals with LBP. The moderate effect observed in this review suggests that MI may help people with LBP initiate and maintain active ­behaviors, potentially supporting broader self-management. Nevertheless, MI was not associated with improvements in pain or disability-related outcomes, indicating that behavior change alone may be insufficient to influence symptom severity or functional capacity. Motivational interviewing may therefore be best conceptualized as a behavior change facilitator within comprehensive rehabilitation models rather than a stand-alone intervention targeting symptom reduction. Future research should examine whether integrating MI with interventions that directly address pain mechanisms and functional limitations results in synergistic improvements in both PA and symptom-related outcomes. Well-designed trials with longer follow-up, robust fidelity assessment, and clear intervention specification are needed to clarify when, for whom, and how MI is most effective.

## Supplementary Material

kaag040_Supplementary_Data

## Data Availability

All data generated or analyzed during this study are included in this published article.

## Transparency statements

For each of the 5 transparency practices, you will provide a statement that accurately describes your article. The following examples may be adapted for use. If none of the statements accurately describe your article, please provide a statement. **Statements should be provided on the cover page and will be published with your manuscript.** 

1. **Study registration**. Study registration involves declaring the study design, variables, and treatment conditions in an independent, institutional repository (eg, http://clinicaltrials.gov, https://osf.io/, https://www.socialscienceregistry.org/*)*. A study is considered *pre*-registered if it is registered in a repository prior to the study began. Consider the following examples:
  - ⚬The study was pre-registered at PROSPERO (registration ID: CRD42024585036)
  - ⚬This study was registered after the study began. The study is registered at _____________________________ (*insert name of repository and link to study registration*).
  - ⚬This study was not formally registered.
2. **Analytic plan pre-registration**. Pre-registration of an analytic plan involves specification of the planned sequence of analyses or the statistical model that will be reported. Reporting the planned outcome variables alone does not qualify as analytic plan pre-registration. Consider the following examples:
  - ⚬The analysis plan was registered prior to beginning data collection at ____________ (*insert name of repository and link to study registration*).
  - ⚬The analysis plan was not formally pre-registered.
3. **Data availability:** Indicate whether the data underlying the presented analyses will be made available in a third-party archive/repository. If data is available in an archive, indicate if this is fully available to the public or has protected access (available for limited audience, with clearly defined requirements and steps to obtain data). If data is not in a public archive, describe any steps needed to access the data. Consider the following examples:
  - ⚬De-identified data from this study are available in a public archive: __________ (*provide url*).
  - ⚬De-identified data from this study are available in a protected archive: _________________________ (*provide url*). Data can be obtained by _________________ (*briefly describe steps and requirements to access data*)
  - ⚬De-identified data from this study are not available in a public archive. De-identified data from this study will be made available (as allowable according to institutional IRB standards) by emailing the corresponding author.
4. **Analytic code availability.** Analytic code refers to the programming code used to conduct the analyses in the statistical software. Consider the following examples:
  - ⚬Analytic code used to conduct the analyses presented in this study are available in a public archive: _____________ (*provide url*)
  - ⚬Analytic code used to conduct the analyses presented in this study are not available in a public archive. They may be available by emailing the corresponding author.
  - ⚬There is not analytic code associated with this study.
5. **Materials availability.** Materials include any stimuli used in this study, intervention protocols, and survey instruments and items. Consider the following examples:
  - ⚬All materials used to conduct the a study are available in a public archive: ____________ (*provide url*).
  - ⚬Some of the materials used to conduct the study are presented in a public archive: ____________ (*provide url*).
  - ⚬Materials used to conduct the study are not publicly available.
