# Supplementary material for: Motivational interviewing increases physical activity for adults with low back pain: a systematic review and meta-analysis
Source: Ann Behav Med. 2026 Sep 2;60(1):kaag040. doi: 10.1093/abm/kaag040 (PMC13537531; doi:10.1093/abm/kaag040)

Electronic supplementary material

Supplementary material S1. PRISMA checklist

Section and Topic	Item #	Checklist item	Location where item is reported
**TITLE**			
Title	1	Identify the report as a systematic review.	1
**ABSTRACT**			
Abstract	2	See the PRISMA 2020 for Abstracts checklist.	2-3
**INTRODUCTION**			
Rationale	3	Describe the rationale for the review in the context of existing knowledge.	4-6
Objectives	4	Provide an explicit statement of the objective(s) or question(s) the review addresses.	6
**METHODS**			
Eligibility criteria	5	Specify the inclusion and exclusion criteria for the review and how studies were grouped for the syntheses.	7-8
Information sources	6	Specify all databases, registers, websites, organisations, reference lists and other sources searched or consulted to identify studies. Specify the date when each source was last searched or consulted.	6-7
Search strategy	7	Present the full search strategies for all databases, registers and websites, including any filters and limits used.	Supp 2
Selection process	8	Specify the methods used to decide whether a study met the inclusion criteria of the review, including how many reviewers screened each record and each report retrieved, whether they worked independently, and if applicable, details of automation tools used in the process.	7-8
Data collection process	9	Specify the methods used to collect data from reports, including how many reviewers collected data from each report, whether they worked independently, any processes for obtaining or confirming data from study investigators, and if applicable, details of automation tools used in the process.	8-9
Data items	10a	List and define all outcomes for which data were sought. Specify whether all results that were compatible with each outcome domain in each study were sought (e.g. for all measures, time points, analyses), and if not, the methods used to decide which results to collect.	8-9
	10b	List and define all other variables for which data were sought (e.g. participant and intervention characteristics, funding sources). Describe any assumptions made about any missing or unclear information.	8-9
Study risk of bias assessment	11	Specify the methods used to assess risk of bias in the included studies, including details of the tool(s) used, how many reviewers assessed each study and whether they worked independently, and if applicable, details of automation tools used in the process.	9
Effect measures	12	Specify for each outcome the effect measure(s) (e.g. risk ratio, mean difference) used in the synthesis or presentation of results.	9 - 10
Synthesis methods	13a	Describe the processes used to decide which studies were eligible for each synthesis (e.g. tabulating the study intervention characteristics and comparing against the planned groups for each synthesis (item #5)).	10
	13b	Describe any methods required to prepare the data for presentation or synthesis, such as handling of missing summary statistics, or data conversions.	10
	13c	Describe any methods used to tabulate or visually display results of individual studies and syntheses.	9 -10
	13d	Describe any methods used to synthesize results and provide a rationale for the choice(s). If meta-analysis was performed, describe the model(s), method(s) to identify the presence and extent of statistical heterogeneity, and software package(s) used.	9 -10
	13e	Describe any methods used to explore possible causes of heterogeneity among study results (e.g. subgroup analysis, meta-regression).	9 - 10
	13f	Describe any sensitivity analyses conducted to assess robustness of the synthesized results.	9 - 10

Section and Topic	Item #	Checklist item	Location where item is reported
Reporting bias assessment	14	Describe any methods used to assess risk of bias due to missing results in a synthesis (arising from reporting biases).	9
Certainty assessment	15	Describe any methods used to assess certainty (or confidence) in the body of evidence for an outcome.	9
**RESULTS**			
Study selection	16a	Describe the results of the search and selection process, from the number of records identified in the search to the number of studies included in the review, ideally using a flow diagram.	12
	16b	Cite studies that might appear to meet the inclusion criteria, but which were excluded, and explain why they were excluded.	12
Study characteristics	17	Cite each included study and present its characteristics.	13-14
Risk of bias in studies	18	Present assessments of risk of bias for each included study.	15
Results of individual studies	19	For all outcomes, present, for each study: (a) summary statistics for each group (where appropriate) and (b) an effect estimate and its precision (e.g. confidence/credible interval), ideally using structured tables or plots.	16-18
Results of syntheses	20a	For each synthesis, briefly summarise the characteristics and risk of bias among contributing studies.	16-18
	20b	Present results of all statistical syntheses conducted. If meta-analysis was done, present for each the summary estimate and its precision (e.g. confidence/credible interval) and measures of statistical heterogeneity. If comparing groups, describe the direction of the effect.	16-18
	20c	Present results of all investigations of possible causes of heterogeneity among study results.	16-18
	20d	Present results of all sensitivity analyses conducted to assess the robustness of the synthesized results.	16-18
Reporting biases	21	Present assessments of risk of bias due to missing results (arising from reporting biases) for each synthesis assessed.	16-18
Certainty of evidence	22	Present assessments of certainty (or confidence) in the body of evidence for each outcome assessed.	16-18
**DISCUSSION**			
Discussion	23a	Provide a general interpretation of the results in the context of other evidence.	18-220
	23b	Discuss any limitations of the evidence included in the review.	18-220
	23c	Discuss any limitations of the review processes used.	18-220
	23d	Discuss implications of the results for practice, policy, and future research.	18-220
**OTHER INFORMATION**			
Registration and protocol	24a	Provide registration information for the review, including register name and registration number, or state that the review was not registered.	6
	24b	Indicate where the review protocol can be accessed, or state that a protocol was not prepared.	6
	24c	Describe and explain any amendments to information provided at registration or in the protocol.	7, 10
Support	25	Describe sources of financial or non-financial support for the review, and the role of the funders or sponsors in the review.	Title page
Competing interests	26	Declare any competing interests of review authors.	Title page
Availability of data, code and	27	Report which of the following are publicly available and where they can be found: template data collection forms; data extracted from included studies; data used for all analyses; analytic code; any other materials used in the review.	Title page

Section and Topic	Item #	Checklist item	Location where item is reported
other materials			

From: Page MJ, McKenzie JE, Bossuyt PM, Boutron I, Hoffmann TC, Mulrow CD, et al. The PRISMA 2020 statement: an updated guideline for reporting systematic reviews. BMJ 2021;372:n71. doi: 10.1136/bmj.n71.
This work is licensed under CC BY 4.0. To view a copy of this license, visit https://creativecommons.org/licenses/by/4.0/

Supplementary Material S2. Detailed search strategies.

MEDLINE
Database: Ovid MEDLINE(R) ALL <1946 to June, 2025>
Search Strategy:

1	motivational interviewing/ or motivat* inter*.mp.	8344
2	motivation/	85684
3	psychological counseling/ or counseling/ or counseling.mp.	133163
4	health behavior/ or health behav*.mp.	84392
5	health coach*.mp.	1752
6	low back pain.mp. or low back pain/	47757
7	lumbar pain.mp. or low back pain/	30861
8	musculoskeletal pain.mp. or pain/ or musculoskeletal pain/ or chronic pain/	197951
9	spinal osteoarthritis/ or osteoarthritis.mp. or osteoarthritis/	127876
10	1 or 2 or 3 or 4 or 5	300400
11	6 or 7 or 8 or 9	359400
12	10 and 11	2948
13	physical activity.mp. or physical activity/	175253
14	muscle exercise/ or exercise.mp. or exercise/ or exercise recovery/	477347
15	walking.mp. or walking/	116791
16	physical fitness.mp. or fitness/	38695
17	13 or 14 or 15 or 16	658460
18	12 and 17	678
19	randomised controlled trial.mp. or randomized controlled trial/	657255
20	18 and 19	143

Embase
Database: Embase Classic <1947 to 1973> Embase <1974 to May 03, 2025>
Search Strategy:

Embase Classic+Embase <1947 to May 03, 2025>

1 motivational interviewing/ or motivat* inter*.mp. 14993
2 motivation/ 147674
3 psychological counseling/ or counseling/ or counseling.mp. 268575
4 health behavior/ or health behav*.mp. 108729
5 health coach*.mp. 3086
6 low back pain.mp. or low back pain/ 96462
7 lumbar pain.mp. or low back pain/ 87716
8 musculoskeletal pain.mp. or pain/ or musculoskeletal pain/ or chronic pain/ 552338
9 spinal osteoarthritis/ or osteoarthritis.mp. or osteoarthritis/ 219242
10 1 or 2 or 3 or 4 or 5 521151
11 6 or 7 or 8 or 9 820541
12 10 and 11 11827
13 physical activity.mp. or physical activity/ 358382
14 muscle exercise/ or exercise.mp. or exercise/ or exercise recovery/ 749836
15 walking.mp. or walking/ 209563
16 physical fitness.mp. or fitness/ 60376
17 13 or 14 or 15 or 16 1153989
18 12 and 17 2945
19 randomised controlled trial.mp. or randomized controlled trial/ 1097617
20 18 and 19 631

Psychinfo
APA PsycInfo <1806 to June 2025

1 motivational interviewing/ or motivat* inter*.mp. 7591
2 motivation/ 68583
3 psychological counseling/ or counseling/ or counseling.mp. 115569

4	health behavior/ or health behav*.mp.	56609
5	health coach*.mp.	484
6	low back pain.mp. or low back pain/	5121
7	lumbar pain.mp. or low back pain/	71
8	musculoskeletal pain.mp. or pain/ or musculoskeletal pain/ or chronic pain/	51586
9	spinal osteoarthritis/ or osteoarthritis.mp. or osteoarthritis/	2840
10	1 or 2 or 3 or 4 or 5	242060
11	6 or 7 or 8 or 9	56065
12	10 and 11	1230
13	physical activity.mp. or physical activity/	56172
14	muscle exercise/ or exercise.mp. or exercise/ or exercise recovery/	83758
15	walking.mp. or walking/	23671
16	physical fitness.mp. or fitness/	9123
17	13 or 14 or 15 or 16	136045
18	12 and 17	208
19	randomised controlled trial.mp. or randomized controlled trial/	6652
20	18 and 19	2
21	motivational interviewing/ or motivat* inter*.mp.	7591
22	motivation/	68583
23	psychological counseling/ or counseling/ or counseling.mp.	115569
24	health behavior/ or health behav*.mp.	56609
25	health coach*.mp.	484
26	low back pain.mp. or low back pain/	5121
27	lumbar pain.mp. or low back pain/	71
28	musculoskeletal pain.mp. or pain/ or musculoskeletal pain/ or chronic pain/	51586
29	spinal osteoarthritis/ or osteoarthritis.mp. or osteoarthritis/	2840
30	21 or 22 or 23 or 24 or 25	242060
31	26 or 27 or 28 or 29	56065
32	30 and 31	1230
33	physical activity.mp. or physical activity/	56172
34	muscle exercise/ or exercise.mp. or exercise/ or exercise recovery/	83758
35	walking.mp. or walking/	23671
36	physical fitness.mp. or fitness/	9123
37	33 or 34 or 35 or 36	136045
38	32 and 37	208

CINAHL to June 01, 2025>

S1	motivational interviewing
S2	motiv* interv*
S3	motivation
S4	counseling or therapy or psychotherapy or treatment
S5	health behavior change
S6	health behav* change
S7	self-management or self-care or self-regulation or self-monitoring
S8	low back pain or lumbar pain or lumbar spine pain or non specific low back pain
S9	lumbar pain
S10	spine pain
S11	musculoskeletal disorders or musculoskeletal pain or musculoskeletal injuries
S12	musculoskeletal disorders or musculo* pain or musculo* injuries
S13	chronic pain or persistent pain or long term pain
S14	exercise or physical fitness or physical activity
S15	physiotherapy or physical therapy or rehabilitation
S16	walking program or walking intervention or walking
S17	motor activity
S18	movement
S19	S1 OR S2 OR S3 OR S4 OR S5 OR S6 OR S7 OR S8
S20	S8 OR S9 OR S10 OR S11 OR S12 OR S13
S21	S14 OR S15 OR S16 OR S17 OR S18
S22	S19 AND S20
S23	S21 AND S22
S24	randomized controlled trials or rtc or randomised control trials
S25	S23 AND S24

Supplementary Material S3.

Item	Sequence generation	Allocation concealment	Blinding of participants and personnel	Blinding of outcome assessment	Incomplete outcome data	Selective reporting	Other bias
**Amorim, 2019**	Low	Low	High	Low	Low	Low	Low
**Ben-Ami, 2017**	Unclear	Low	High	Low	Low	Low	Low
**Holden, 2024**	Low	Low	Unclear	Low	Low	Low	Low
**Iles, 2011**	Low	Low	High	Low	Low	Low	Low
**Leonhardt, 2008**	Low	Unclear	High	Unclear	Low	Low	Low
**Rodrigues, 2021**	Unclear	Unclear	Unclear	Unclear	Low	Low	Low
**Shimo, 2021**	Low	Low	Unclear	Unclear	Low	Low	Low
**Vong, 2011**	Low	Low	Low	Low	Low	Low	Low

Supplementary Material S4. Excluded studies from full text review

Aboagye 2015	Cost-effectiveness of early interventions for non-specific low back pain: a randomized controlled study investigating medical yoga, exercise therapy and self-care advice	Wrong study design
Buus 2015	Low back pain patients' beliefs about effective/ineffective constituents of a counseling intervention: A follow-up interview study.	Wrong study design
Makris 2021	Improving outcomes in older veterans with chronic low back pain and comorbid depression: Preliminary data from the motivate pilot trial	Wrong study design
NCT04257539 2020	Reducing Sedentary Time in Chronic Low Back Pain: sedentary Intervention Using Motivational Interviewing and Technology	Wrong study design
Oliveira 2018	The efficacy of a multimodal physical activity intervention with supervised exercises, health coaching and an activity monitor on physical activity levels of patients with chronic, nonspecific low back pain (Physical Activity for Back Pain (PAyBACK) trial	Wrong study design
Robson 2022	Compliance with telephone-based lifestyle weight loss programs improves low back pain but not knee pain outcomes: Complier average causal effects analyses of 2 randomised trials	Wrong study design
Williams 2019	Economic evaluation of a healthy lifestyle intervention for chronic low back pain: A randomized controlled trial.	Wrong study design
Jay 2015	Effect of Individually Tailored Biopsychosocial Workplace Interventions on Chronic Musculoskeletal Pain and Stress Among Laboratory Technicians: Randomized Controlled Trial.	Wrong population
Katalin Forgács-Kristóf 2024	Novel motivational interviewing-based intervention improves engagement in physical activity and readiness to change among adolescents with chronic pain	Wrong population
Cheing 2014	Testing a path-analytic mediation model of how motivational enhancement physiotherapy improves physical functioning in pain patients	Wrong outcomes
Hergenroeder 2022	Effect of a 6-month sedentary behavior reduction intervention on well-being and workplace health in desk workers with low back pain.	Wrong outcomes
Hunt 2013	A physiotherapist-delivered, combined exercise and pain coping skills training intervention for individuals with knee osteoarthritis: A pilot study	Wrong outcomes
Igwesi-Chidobe 2019	Community-based self-management of chronic low back pain in a rural African primary care setting: A feasibility study.	Wrong outcomes
Kasimis 2024	Effects of Manual Therapy Plus Pain Neuroscience Education with Integrated Motivational Interviewing in Individuals with Chronic Non-Specific Low Back Pain: A Randomized Clinical Trial Study	Wrong outcomes
ACTRN12618001628235 2018	CONNECT: telecare health coaching management of low back pain in primary care to improve disability	Wrong intervention
Beekman 1985	Self-concept: an outcome of a program for spinal pain.	Wrong intervention

Burtin 1999	Information and advice to patients with back pain can have a positive effect. A randomized controlled trial of a novel educational booklet in primary care.	Wrong intervention
Coppack 2012	Use of a goal setting intervention to increase adherence to low back pain rehabilitation: a randomized controlled trial.	Wrong intervention
Cui 2023	Randomized-controlled trial assessing a digital care program versus conventional physiotherapy for chronic low back pain.	Wrong intervention
deCampos 2020	An individualised self-management exercise and education program did not prevent recurrence of low back pain but may reduce care seeking: a randomised trial.	Wrong intervention
delPozo-Cruz 2012	An occupational, internet-based intervention to prevent chronicity in subacute lower back pain: a randomized controlled trial.	Wrong intervention
delPozo-Cruz 2013	Clinical effects of a nine-month web-based intervention in subacute non-specific low back pain patients: a randomized controlled trial.	Wrong intervention
Delshad 2024	The effectiveness of a theory-based educational intervention on promoting physical activity-related behavior in patients with chronic low back pain: a randomized controlled trial based on health center (TRA -BAC)	Wrong intervention
Ersek 2008	Results of a randomized controlled trial to examine the efficacy of a chronic pain self-management group for older adults [ISRCTN11899548].	Wrong intervention
Friedrich 1998	Combined exercise and motivation program: effect on the compliance and level of disability of patients with chronic low back pain: a randomized controlled trial.	Wrong intervention
Geraghty 2018	Using an internet intervention to support self-management of low back pain in primary care: findings from a randomised controlled feasibility trial (SupportBack).	Wrong intervention
Godges 2008	Effects of education on return-to-work status for people with fear-avoidance beliefs and acute low back pain.	Wrong intervention
Goode 2018	Effects of a Home-Based Telephone-Supported Physical Activity Program for Older Adult Veterans With Chronic Low Back Pain.	Wrong intervention
Hirase 2018	Effects of a psychosocial intervention programme combined with exercise in community-dwelling older adults with chronic pain: A randomized controlled trial.	Wrong intervention
In 2021	Effects of the Multidimensional Treatment on Pain, Disability, and Sitting Posture in Patients with Low Back Pain: A Randomized Controlled Trial.	Wrong intervention
Jensen 2012	Counselling low-back-pain patients in secondary healthcare: a randomised trial addressing experienced workplace barriers and physical activity.	Wrong intervention
Langagergaard 2021	The comparative effects of brief or multidisciplinary intervention on return to work at 1â€‰year in employees on sick leave due to low back pain: A randomized controlled trial.	Wrong intervention
Leonhardt 2008	TTM-based motivational counseling does not increase physical activity of low back pain patients in a primary care setting--A cluster-randomized controlled trial.	Wrong intervention

Lynch 2018	Individuals with lumbar spinal stenosis seek education and care focused on self-management-results of focus groups among participants enrolled in a randomized controlled trial.	Wrong intervention
MachadoLAC 2007	Client-Centered Therapy vs Exercise Therapy for Chronic Low Back Pain: A Pilot Randomized Controlled Trial in Brazil.	Wrong intervention
MatarÃ¡n-PeÃ±arrocha 2020	Comparison of efficacy of a supervised versus non-supervised physical therapy exercise program on the pain, functionality and quality of life of patients with non-specific chronic low-back pain: a randomized controlled trial.	Wrong intervention
Roche 2007	Comparison of a functional restoration program with active individual physical therapy for patients with chronic low back pain: a randomized controlled trial.	Wrong intervention
Rotter 2022	Mindful Walking in Patients with Chronic Low Back Pain: A Randomized Controlled Trial.	Wrong intervention
Saracoglu 2022	The effectiveness of pain neuroscience education combined with manual therapy and home exercise for chronic low back pain: A single-blind randomized controlled trial.	Wrong intervention
Schaller 2017	Effectiveness of a theory-based multicomponent intervention (Movement Coaching) on the promotion of total and domain-specific physical activity: a randomised controlled trial in low back pain patients.	Wrong intervention
Semrau 2021	Effects of behavioural exercise therapy on the effectiveness of multidisciplinary rehabilitation for chronic non-specific low back pain: a randomised controlled trial.	Wrong intervention
Siemonsma 2013	Cognitive Treatment of Illness Perceptions in Patients With Chronic Low Back Pain: A Randomized Controlled Trial.	Wrong intervention
SmeetsRJE 2006	Reduction of pain catastrophizing mediates the outcome of both physical and cognitive-behavioral treatment in chronic low back pain.	Wrong intervention
Suni 2018	Effectiveness and cost-effectiveness of neuromuscular exercise and back care counseling in female healthcare workers with recurrent non-specific low back pain: a blinded four-arm randomized controlled trial.	Wrong intervention
Tavafian 2008	A randomized study of back school in women with chronic low back pain: quality of life at three, six, and twelve months follow-up.	Wrong intervention
Wessels 2007	Change factors explaining reductions of 'interference' in a multidisciplinary and an exercise prevention program for low back pain.	Wrong intervention

Supplementary Material S5. Subgroup analysis of changes in physical activity by pain duration

Study or Subgroup	Experimental Mean	Experimental SD	Experimental Total	Control Mean	Control SD	Control Total	Weight	Std. mean difference IV, Random, 95% CI
**2.4.1 Chronic pain**								
Amorin, 2019	187.7	138.5	34	169.2	131.8	31	12.9%	0.14 [-0.35 , 0.62]
Ben-Ami, 2017	0.8	2.3	94	0.1	2.2	95	14.9%	0.31 [0.02 , 0.60]
Rordigues, 2021	92.1	11.8	30	75.9	15.5	30	12.2%	1.16 [0.61 , 1.71]
Shimo, 2021	12694	3082	20	9429	2903	17	10.6%	1.06 [0.37 , 1.76]
Vong, 2011	12.9	7.2	38	5.8	4.1	38	12.8%	1.20 [0.71 , 1.69]
**Subtotal (HKSJ[a])**			**216**			**211**	**63.4%**	**0.74 [0.10 , 1.38]**
Test for overall effect: T = 3.22, df = 4 (P = 0.03)								
Heterogeneity: Tau² (REML[b]) = 0.21; Chi² = 18.87, df = 4 (P = 0.0008); I² = 78%								
**2.4.2 Acute/Sub-acute**								
Holden, 2024	9.7	9.6	21	11.7	16.1	19	11.4%	-0.15 [-0.77 , 0.47]
Iles, 2011	8.3	2.1	13	5.2	3.4	13	9.3%	1.06 [0.23 , 1.89]
Leonhardt, 2018	45.5	38.8	489	42.7	17.7	410	15.9%	0.09 [-0.04 , 0.22]
**Subtotal (HKSJ[a])**			**523**			**442**	**36.6%**	**0.25 [-1.18 , 1.68]**
Test for overall effect: T = 0.76, df = 2 (P = 0.53)								
Heterogeneity: Tau² (REML[b]) = 0.20; Chi² = 5.80, df = 2 (P = 0.05); I² = 75%								
**Total (HKSJ[a])**			**739**			**653**	**100.0%**	**0.57 [0.10 , 1.03]**

Test for overall effect: T = 2.88, df = 7 (P = 0.02)

Test for subgroup differences: Chi² = 1.46, df = 1 (P = 0.23), I² = 31.5%

Heterogeneity: Tau² (REML[b]) = 0.24; Chi² = 41.54, df = 7 (P < 0.00001); I² = 86%

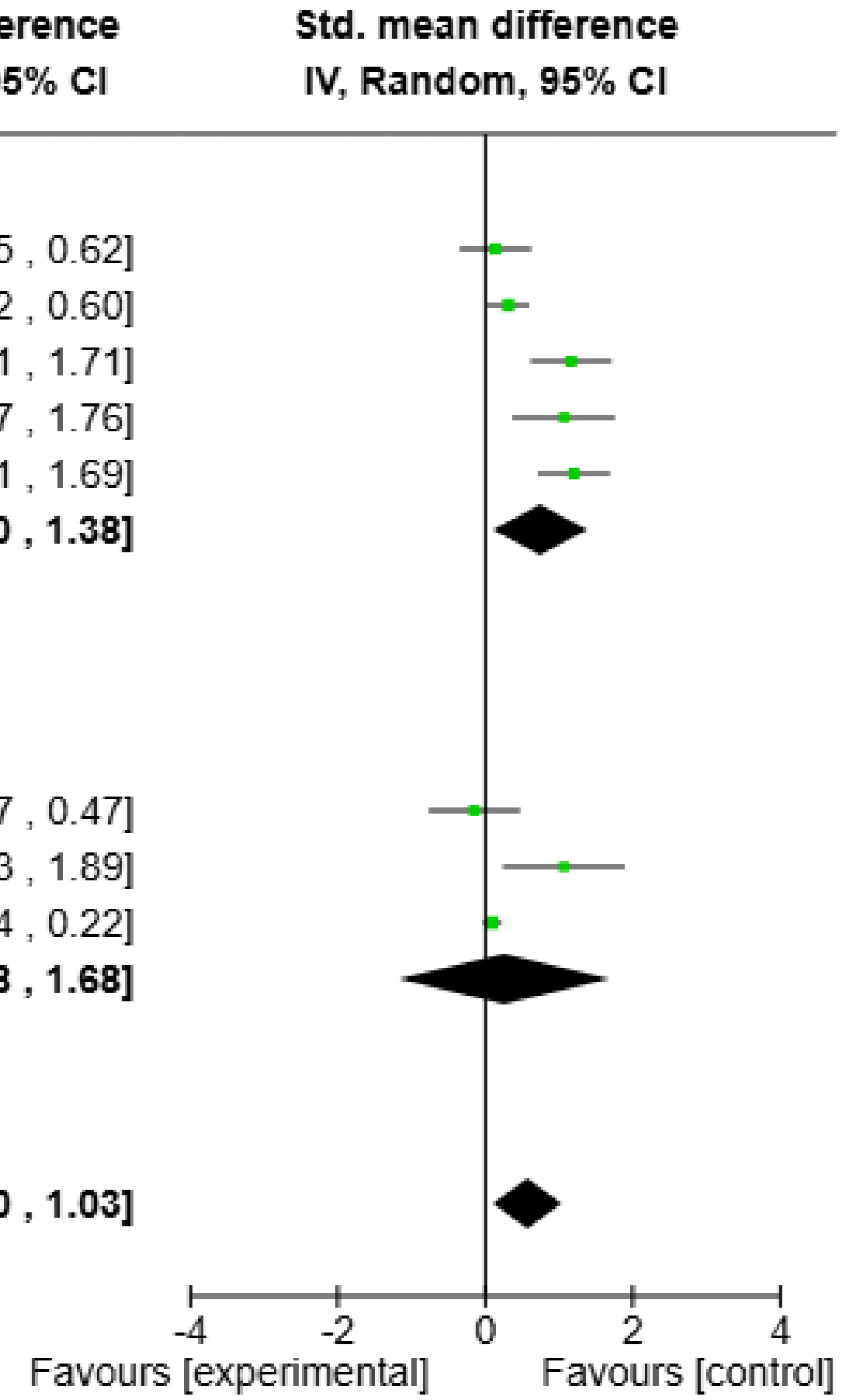

Footnotes

[a]CI calculated by Hartung-Knapp-Sidik-Jonkman method.

[b]Tau² calculated by Restricted Maximum-Likelihood method.

Supplementary Material S6. Subgroup analysis of changes in physical activity by number of sessions

Study or Subgroup	Experimental Mean	Experimental SD	Experimental Total	Control Mean	Control SD	Control Total	Weight	Std. mean difference IV, Random, 95% CI
**2.6.1 ≤ 3 sessions**								
Leonhardt, 2018	45.5	38.8	489	42.7	17.7	410	15.9%	0.09 [-0.04 , 0.22]
Rordigues, 2021	92.1	11.8	30	75.9	15.5	30	12.2%	1.16 [0.61 , 1.71]
**Subtotal (HKSJ[a])**			**519**			**440**	**28.1%**	**0.59 [-6.20 , 7.38]**
Test for overall effect: T = 1.11, df = 1 (P = 0.47)								
Heterogeneity: Tau² (REML[b]) = 0.53; Chi² = 13.78, df = 1 (P = 0.0002); I² = 93%								
**2.6.2 ≥ 4 sessions**								
Amorin, 2019	187.7	138.5	34	169.2	131.8	31	12.9%	0.14 [-0.35 , 0.62]
Ben-Ami, 2017	0.8	2.3	94	0.1	2.2	95	14.9%	0.31 [0.02 , 0.60]
Holden, 2024	9.7	9.6	21	11.7	16.1	19	11.4%	-0.15 [-0.77 , 0.47]
Iles, 2011	8.3	2.1	13	5.2	3.4	13	9.3%	1.06 [0.23 , 1.89]
Shimo, 2021	12694	3082	20	9429	2903	17	10.6%	1.06 [0.37 , 1.76]
Vong, 2011	12.9	7.2	38	5.8	4.1	38	12.8%	1.20 [0.71 , 1.69]
**Subtotal (HKSJ[a])**			**220**			**213**	**71.9%**	**0.57 [-0.02 , 1.17]**
Test for overall effect: T = 2.47, df = 5 (P = 0.06)								
Heterogeneity: Tau² (REML[b]) = 0.23; Chi² = 20.09, df = 5 (P = 0.001); I² = 78%								
**Total (HKSJ[a])**			**739**			**653**	**100.0%**	**0.57 [0.10 , 1.03]**

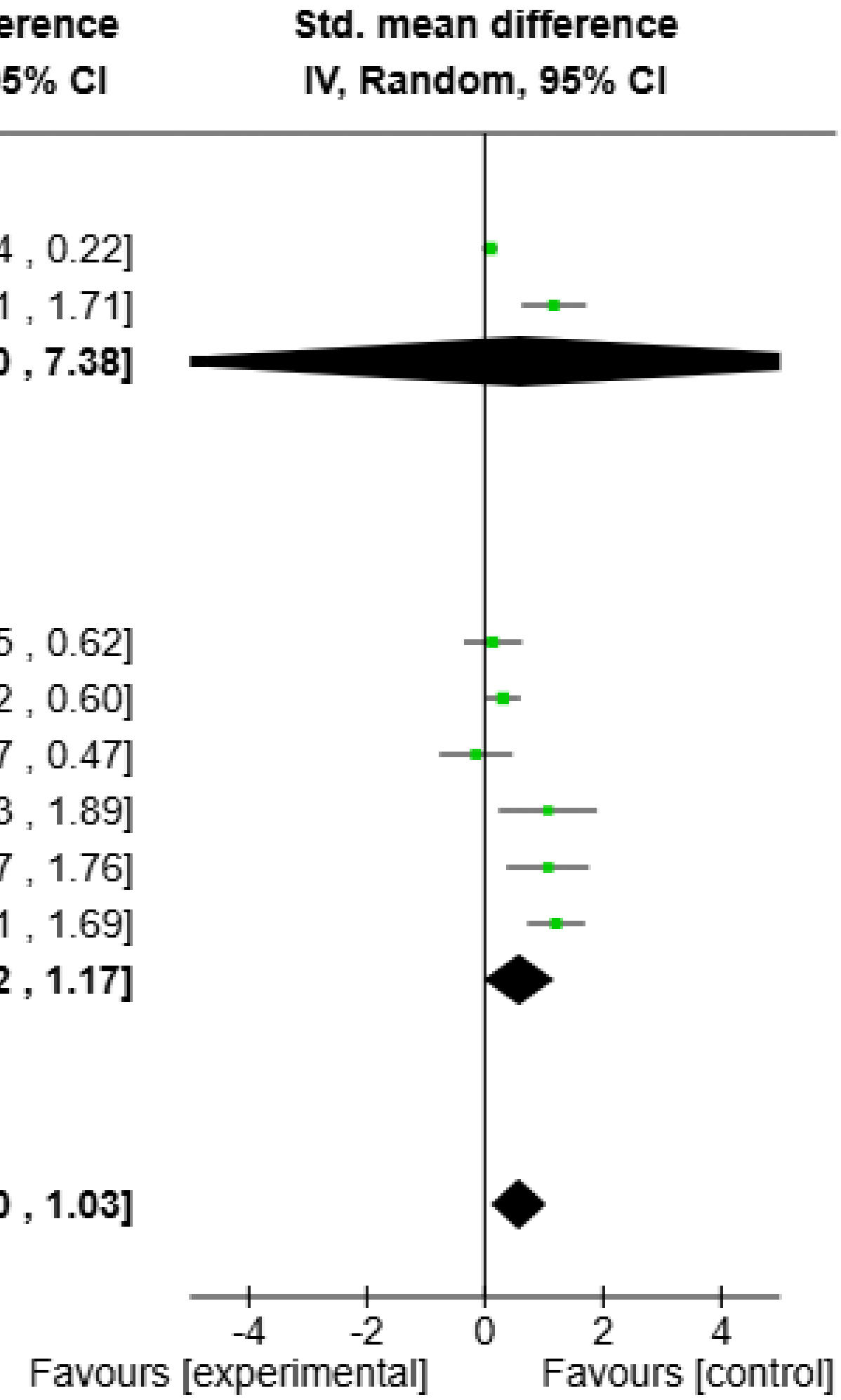

Test for overall effect: T = 2.88, df = 7 (P = 0.02)
Test for subgroup differences: Chi² = 0.00, df = 1 (P = 0.97), I² = 0%
Heterogeneity: Tau² (REML[b]) = 0.24; Chi² = 41.54, df = 7 (P < 0.00001); I² = 86%

Footnotes

[a]CI calculated by Hartung-Knapp-Sidik-Jonkman method.
[b]Tau² calculated by Restricted Maximum-Likelihood method.

Supplementary Material S7. Subgroup analysis of changes in physical activity by comparator type

	Experimental			Control				Std. mean difference
**Study or Subgroup**	**Mean**	**SD**	**Total**	**Mean**	**SD**	**Total**	**Weight**	**IV, Random, 95% CI**
**2.5.1 Active comparator**								
Ben-Ami, 2017	0.8	2.3	94	0.1	2.2	95	14.9%	0.31 [0.02 , 0.60]
Holden, 2024	9.7	9.6	21	11.7	16.1	19	11.4%	-0.15 [-0.77 , 0.47]
Iles, 2011	8.3	2.1	13	5.2	3.4	13	9.3%	1.06 [0.23 , 1.89]
Rordigues, 2021	92.1	11.8	30	75.9	15.5	30	12.2%	1.16 [0.61 , 1.71]
Shimo, 2021	12694	3082	20	9429	2903	17	10.6%	1.06 [0.37 , 1.76]
Vong, 2011	12.9	7.2	38	5.8	4.1	38	12.8%	1.20 [0.71 , 1.69]
**Subtotal (HKSJ[a])**			**216**			**212**	**71.2%**	**0.75 [0.16 , 1.34]**
Test for overall effect: T = 3.28, df = 5 (P = 0.02)								
Heterogeneity: Tau² (REML[b]) = 0.24; Chi² = 22.12, df = 5 (P = 0.0005); I² = 77%								
**2.5.2 No intervention**								
Amorin, 2019	187.7	138.5	34	169.2	131.8	31	12.9%	0.14 [-0.35 , 0.62]
Leonhardt, 2018	45.5	38.8	489	42.7	17.7	410	15.9%	0.09 [-0.04 , 0.22]
**Subtotal (HKSJ[a])**			**523**			**441**	**28.8%**	**0.09 [-0.05 , 0.24]**
Test for overall effect: T = 8.28, df = 1 (P = 0.08)								
Heterogeneity: Tau² (REML[b]) = 0.00; Chi² = 0.03, df = 1 (P = 0.86); I² = 0%								
**Total (HKSJ[a])**			**739**			**653**	**100.0%**	**0.57 [0.10 , 1.03]**

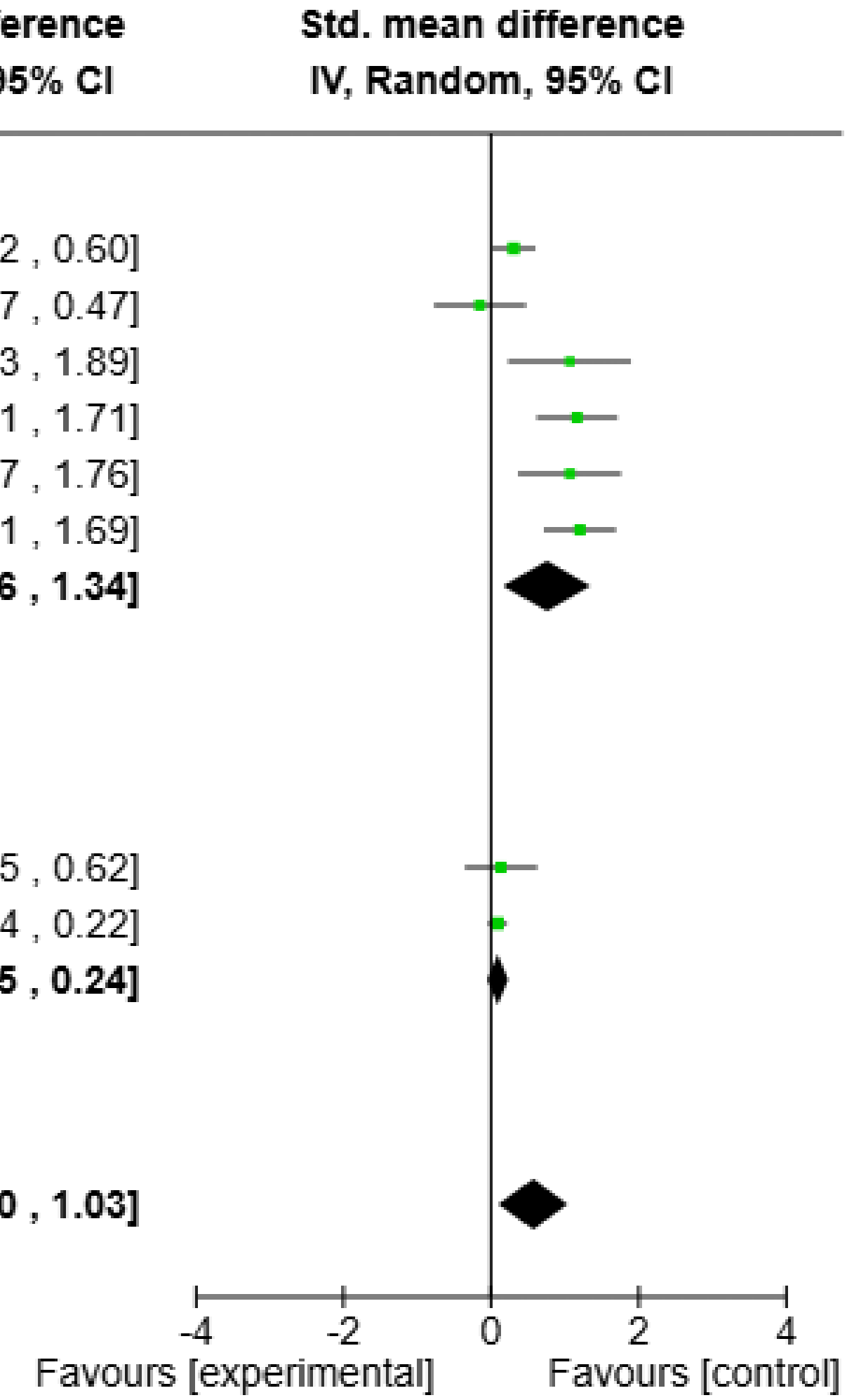

Test for overall effect: T = 2.88, df = 7 (P = 0.02)
Test for subgroup differences: Chi² = 8.24, df = 1 (P = 0.004), I² = 87.9%
Heterogeneity: Tau² (REML[b]) = 0.24; Chi² = 41.54, df = 7 (P < 0.00001); I² = 86%

Footnotes

[a]CI calculated by Hartung-Knapp-Sidik-Jonkman method.
[b]Tau² calculated by Restricted Maximum-Likelihood method.

Supplementary Material S8. Subgroup analysis of changes in physical activity by provider discipline

	Experimental			Control				Std. mean difference
**Study or Subgroup**	**Mean**	**SD**	**Total**	**Mean**	**SD**	**Total**	**Weight**	**IV, Random, 95% CI**
**2.7.1 Physiotherapist**								
Ben-Ami, 2017	0.8	2.3	94	0.1	2.2	95	14.9%	0.31 [0.02 , 0.60]
Holden, 2024	9.7	9.6	21	11.7	16.1	19	11.4%	-0.15 [-0.77 , 0.47]
Iles, 2011	8.3	2.1	13	5.2	3.4	13	9.3%	1.06 [0.23 , 1.89]
Rordigues, 2021	92.1	11.8	30	75.9	15.5	30	12.2%	1.16 [0.61 , 1.71]
Shimo, 2021	12694	3082	20	9429	2903	17	10.6%	1.06 [0.37 , 1.76]
Vong, 2011	12.9	7.2	38	5.8	4.1	38	12.8%	1.20 [0.71 , 1.69]
**Subtotal (HKSJ[a])**			**216**			**212**	**71.2%**	**0.75 [0.16 , 1.34]**
Test for overall effect: T = 3.28, df = 5 (P = 0.02)								
Heterogeneity: Tau² (REML[b]) = 0.24; Chi² = 22.12, df = 5 (P = 0.0005); I² = 77%								
**2.7.2 Other discipline**								
Amorin, 2019	187.7	138.5	34	169.2	131.8	31	12.9%	0.14 [-0.35 , 0.62]
Leonhardt, 2018	45.5	38.8	489	42.7	17.7	410	15.9%	0.09 [-0.04 , 0.22]
**Subtotal (HKSJ[a])**			**523**			**441**	**28.8%**	**0.09 [-0.05 , 0.24]**
Test for overall effect: T = 8.28, df = 1 (P = 0.08)								
Heterogeneity: Tau² (REML[b]) = 0.00; Chi² = 0.03, df = 1 (P = 0.86); I² = 0%								
**Total (HKSJ[a])**			**739**			**653**	**100.0%**	**0.57 [0.10 , 1.03]**

Test for overall effect: T = 2.88, df = 7 (P = 0.02)
Test for subgroup differences: Chi² = 8.24, df = 1 (P = 0.004), I² = 87.9%
Heterogeneity: Tau² (REML[b]) = 0.24; Chi² = 41.54, df = 7 (P < 0.00001); I² = 86%

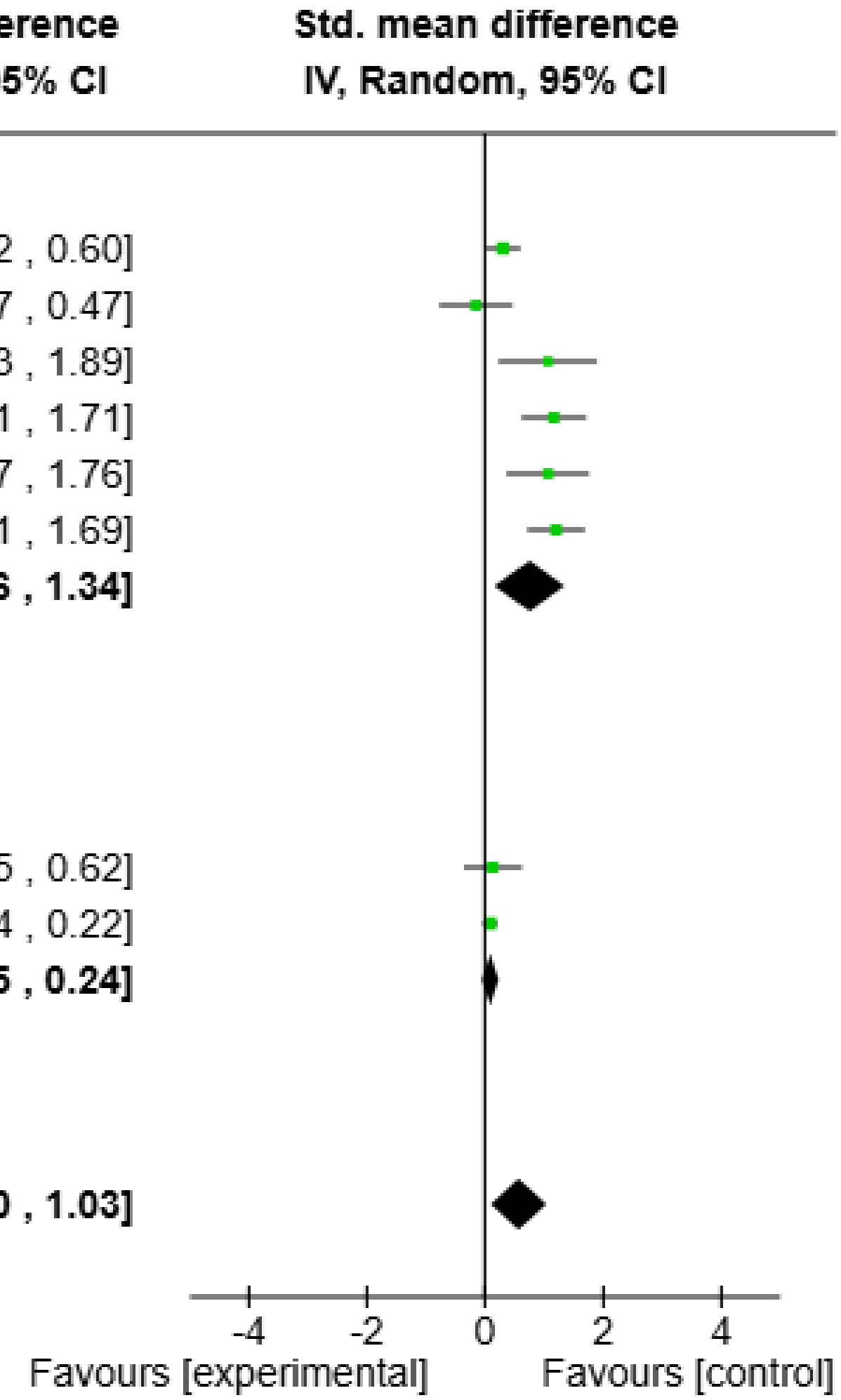

Footnotes

[a]CI calculated by Hartung-Knapp-Sidik-Jonkman method.
[b]Tau² calculated by Restricted Maximum-Likelihood method.

Supplementary Material S9. Subgroup analysis of changes in physical activity by measurement method

Study or Subgroup	Experimental Mean	Experimental SD	Experimental Total	Control Mean	Control SD	Control Total	Weight	Std. mean difference IV, Random, 95% CI
**2.8.1 Device measured**								
Amorin, 2019	187.7	138.5	34	169.2	131.8	31	12.9%	0.14 [-0.35 , 0.62]
Holden, 2024	9.7	9.6	21	11.7	16.1	19	11.4%	-0.15 [-0.77 , 0.47]
Shimo, 2021	12694	3082	20	9429	2903	17	10.6%	1.06 [0.37 , 1.76]
**Subtotal (HKSJ[a])**			**75**			**67**	**34.9%**	**0.33 [-1.21 , 1.86]**
Test for overall effect: T = 0.92, df = 2 (P = 0.46)								
Heterogeneity: Tau² (REML[b]) = 0.27; Chi² = 7.06, df = 2 (P = 0.03); I² = 74%								
**2.8.2 Self-reported**								
Ben-Ami, 2017	0.8	2.3	94	0.1	2.2	95	14.9%	0.31 [0.02 , 0.60]
Iles, 2011	8.3	2.1	13	5.2	3.4	13	9.3%	1.06 [0.23 , 1.89]
Leonhardt, 2018	45.5	38.8	489	42.7	17.7	410	15.9%	0.09 [-0.04 , 0.22]
Rordigues, 2021	92.1	11.8	30	75.9	15.5	30	12.2%	1.16 [0.61 , 1.71]
Vong, 2011	12.9	7.2	38	5.8	4.1	38	12.8%	1.20 [0.71 , 1.69]
**Subtotal (HKSJ[a])**			**664**			**586**	**65.1%**	**0.70 [0.03 , 1.37]**
Test for overall effect: T = 2.91, df = 4 (P = 0.04)								
Heterogeneity: Tau² (REML[b]) = 0.25; Chi² = 34.47, df = 4 (P < 0.00001); I² = 89%								
**Total (HKSJ[a])**			**739**			**653**	**100.0%**	**0.57 [0.10 , 1.03]**

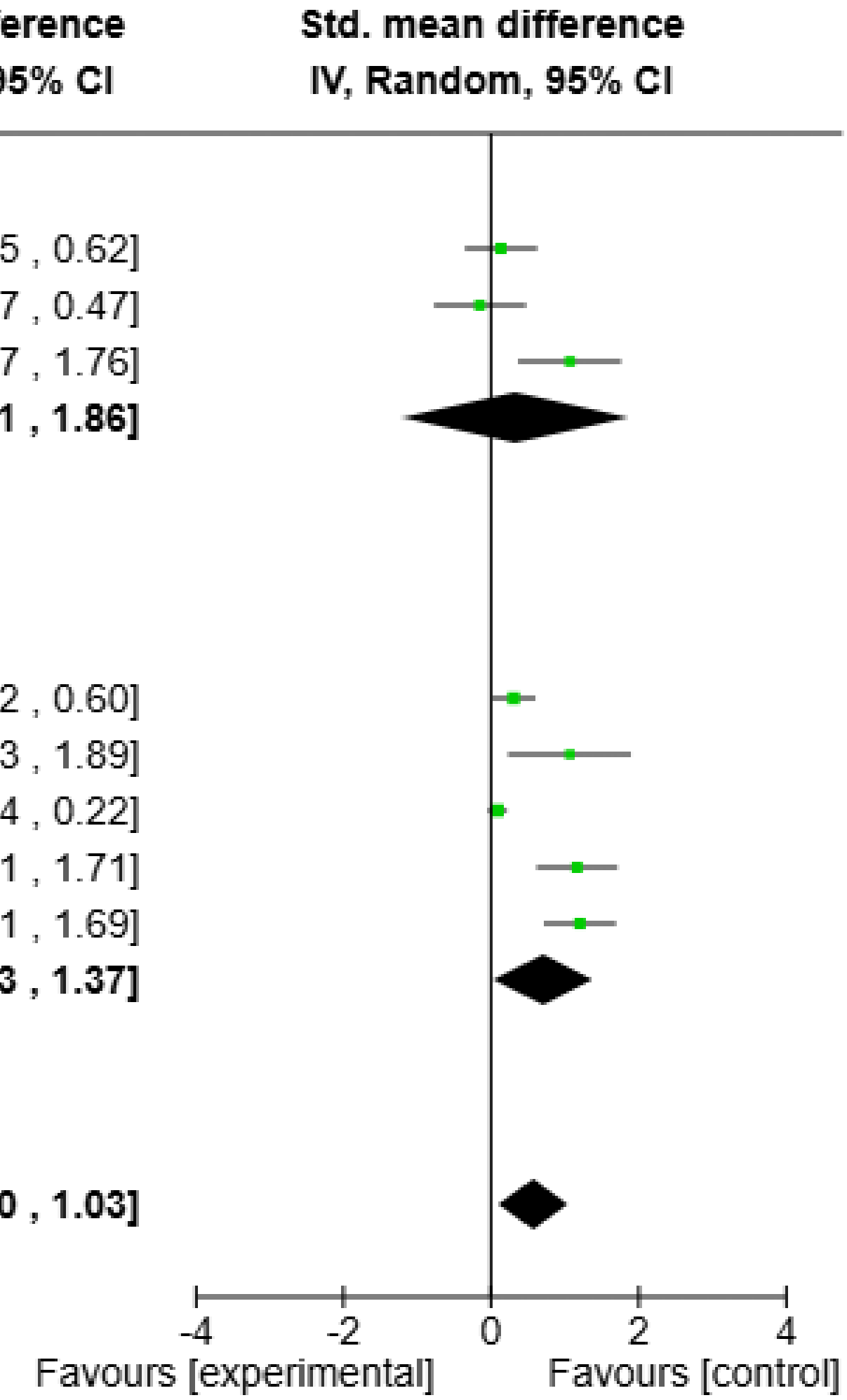

Test for overall effect: T = 2.88, df = 7 (P = 0.02)
Test for subgroup differences: Chi² = 0.76, df = 1 (P = 0.38), I² = 0%
Heterogeneity: Tau² (REML[b]) = 0.24; Chi² = 41.54, df = 7 (P < 0.00001); I² = 86%

Footnotes

[a]CI calculated by Hartung-Knapp-Sidik-Jonkman method.
[b]Tau² calculated by Restricted Maximum-Likelihood method.

Supplementary Material S10: Sensitivity analysis for changes in physical activity excluding high risk of bias studies

Study or Subgroup	Motivational Interviewing Mean	SD	Total	Control Mean	SD	Total	Weight	Std. mean difference IV, Random, 95% CI	Risk of Bias A	B	C	D	E	F
✓ Holden, 2024	9.7	9.6	21	11.7	16.1	19	24.4%	-0.15 [-0.77 , 0.47]	+	+	?	+	+	+
✕ Leonhardt, 2018	45.5	38.8	489	42.7	17.7	410	0.0%	0.09 [-0.04 , 0.22]	+	?	−	+	+	+
✕ Amorin, 2019	187.7	138.5	34	169.2	131.8	31	0.0%	0.14 [-0.35 , 0.62]	+	+	−	+	+	+
✕ Ben-Ami, 2017	0.8	2.3	94	0.1	2.2	95	0.0%	0.31 [0.02 , 0.60]	?	+	−	+	+	+
✕ Iles, 2011	8.3	2.1	13	5.2	3.4	13	0.0%	1.06 [0.23 , 1.89]	+	+	−	+	+	+
✓ Shimo, 2021	12694	3082	20	9429	2903	17	23.0%	1.06 [0.37 , 1.76]	+	+	?	+	+	+
✓ Rordigues, 2021	92.1	11.8	30	75.9	15.5	30	25.7%	1.16 [0.61 , 1.71]	?	?	?	+	+	+
✓ Vong, 2011	12.9	7.2	38	5.8	4.1	38	26.8%	1.20 [0.71 , 1.69]	+	+	+	+	+	+
**Total (HKSJ[a])**			**109**			**104**	**100.0%**	**0.83 [-0.20 , 1.85]**						

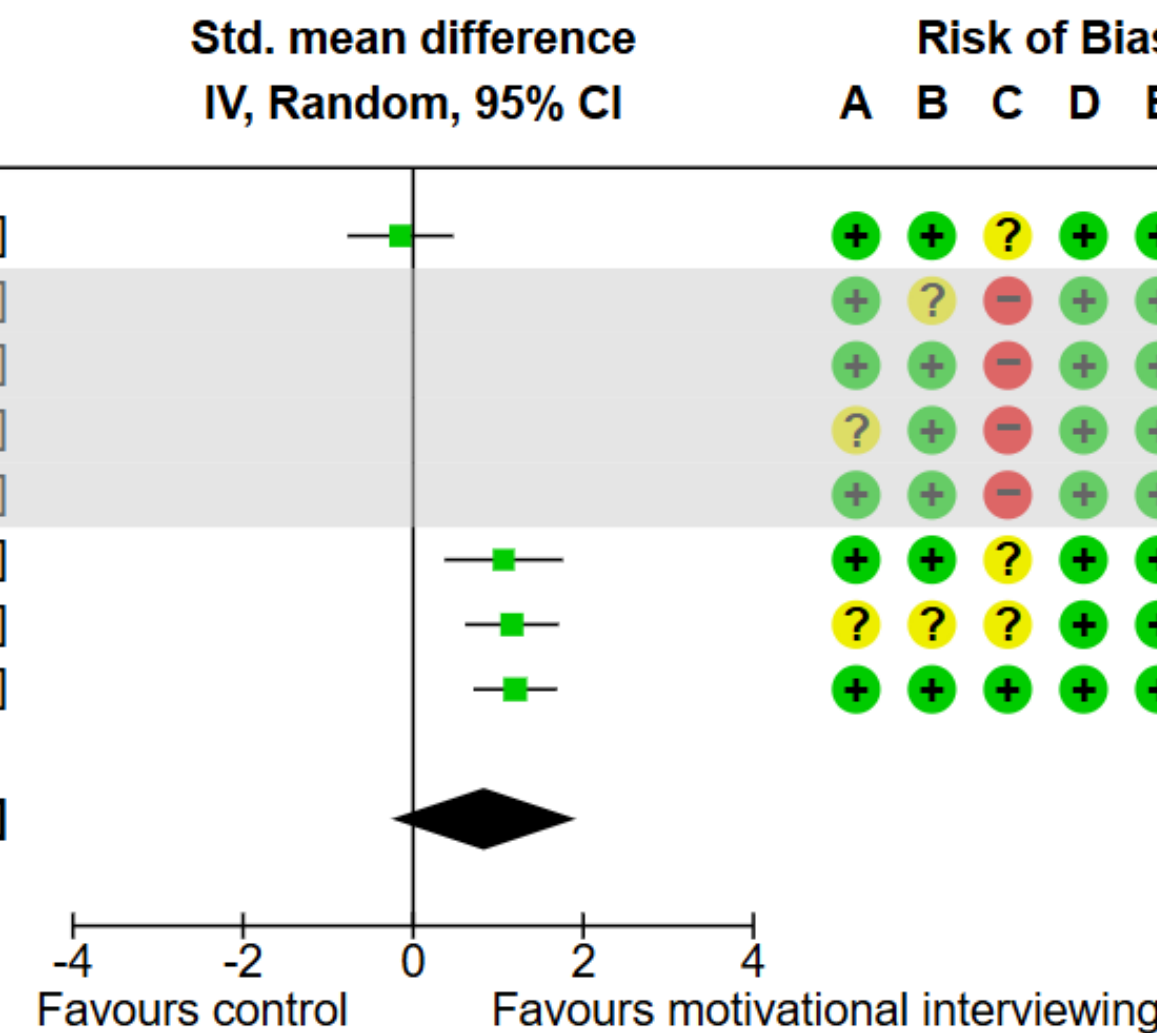

Test for overall effect: T = 2.57, df = 3 (P = 0.08)

Heterogeneity: Tau² (REML[b]) = 0.32; Chi² = 13.45, df = 3 (P = 0.004); I² = 79%

Footnotes

[a]CI calculated by Hartung-Knapp-Sidik-Jonkman method.
[b]Tau² calculated by Restricted Maximum-Likelihood method.

Risk of bias legend

(A) Random sequence generation (selection bias)
(B) Allocation concealment (selection bias)
(C) Blinding of participants and personnel (performance bias)
(D) Incomplete outcome data (attrition bias)
(E) Selective reporting (reporting bias)
(F) Other bias

Supplementary Material S11: Sensitivity analysis for changes in pain excluding high risk of bias studies

Study or Subgroup	Experimental Mean	Experimental SD	Experimental Total	Control Mean	Control SD	Control Total	Weight	Std. mean difference IV, Random, 95% CI	A	B	C	D	E	F
✕ Iles, 2011	8	13	13	18	18	13	0.0%	-0.62 [-1.41 , 0.17]	+	+	−	+	+	+
✓ Vong, 2011	3.1	2.1	38	3.9	2.5	38	28.4%	-0.34 [-0.80 , 0.11]	+	+	+	+	+	+
✕ Leonhardt, 2018	61.6	79.8	489	71.3	78.3	410	0.0%	-0.12 [-0.25 , 0.01]	+	?	−	+	+	+
✕ Amorin, 2019	3.8	2.4	34	4	3.4	31	0.0%	-0.07 [-0.55 , 0.42]	+	+	−	+	+	+
✓ Shimo, 2021	22.6	24.7	20	20.5	15.2	17	22.5%	0.10 [-0.55 , 0.75]	+	+	?	+	+	+
✕ Ben-Ami, 2017	2.6	3.4	94	1.7	3.9	95	0.0%	0.24 [-0.04 , 0.53]	?	+	−	+	+	+
✓ Rordigues, 2021	2.8	0.6	30	2.6	0.5	30	26.6%	0.36 [-0.15 , 0.87]	?	?	?	+	+	+
✓ Holden, 2024	18.2	13	21	8.9	8.7	19	22.5%	0.82 [0.17 , 1.46]	+	+	?	+	+	+
**Total (HKSJ[a])**			**109**			**104**	**100.0%**	**0.20 [-0.57 , 0.98]**						

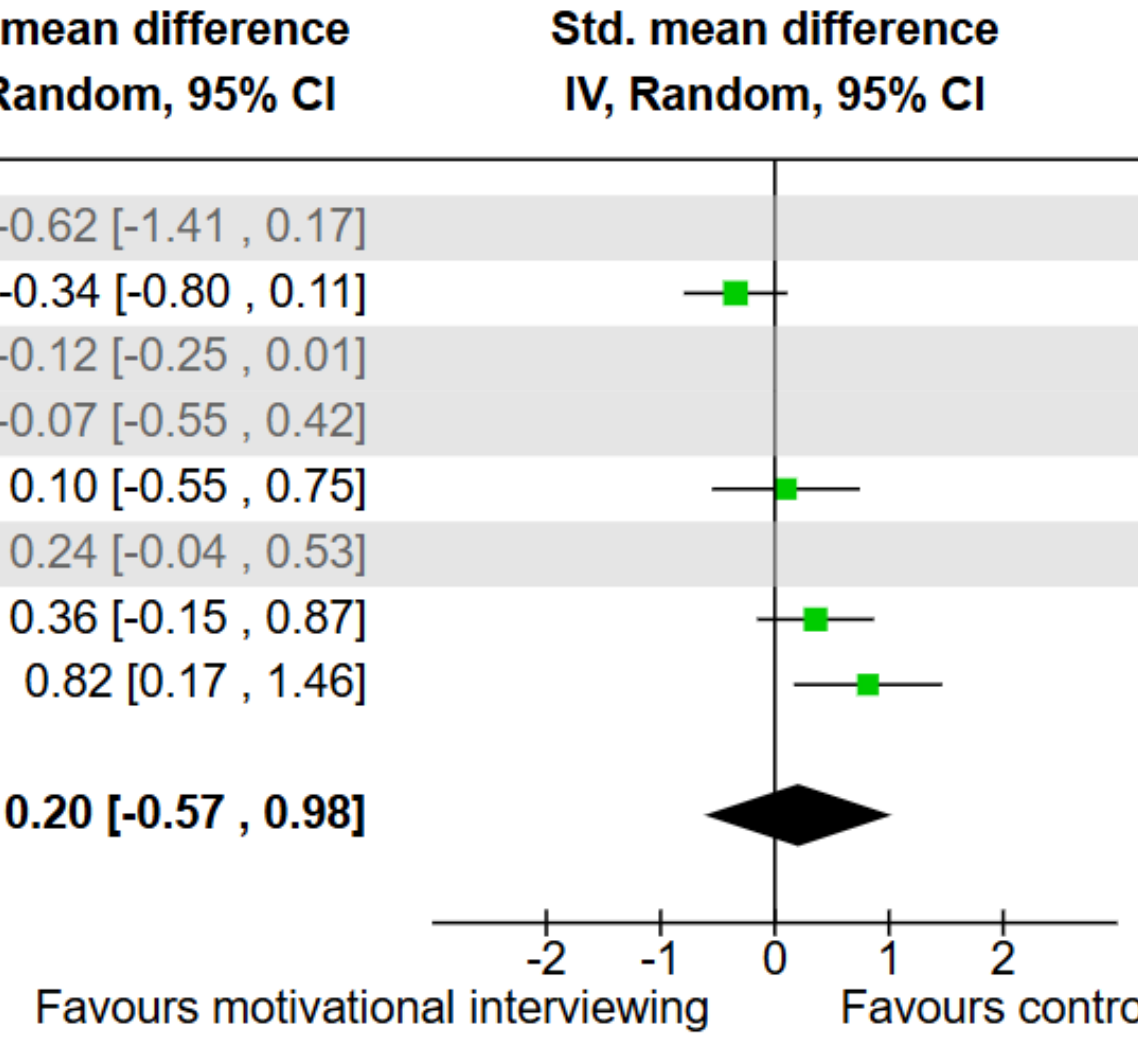

Test for overall effect: T = 0.83, df = 3 (P = 0.47)

Heterogeneity: Tau^2 (REML[b]) = 0.16; Chi^2 = 9.25, df = 3 (P = 0.03); I^2 = 67%

Footnotes

[a]CI calculated by Hartung-Knapp-Sidik-Jonkman method.

[b]Tau^2 calculated by Restricted Maximum-Likelihood method.

Risk of bias legend

(A) Random sequence generation (selection bias)
(B) Allocation concealment (selection bias)
(C) Blinding of participants and personnel (performance bias)
(D) Incomplete outcome data (attrition bias)
(E) Selective reporting (reporting bias)
(F) Other bias

Supplementary Material S12: Sensitivity analysis for changes in disability excluding high risk of bias studies

	Experimental			Control				Std. mean difference	Risk of Bias					
Study or Subgroup	Mean	SD	Total	Mean	SD	Total	Weight	IV, Random, 95% CI	A	B	C	D	E	F
✕ Leonhardt, 2018	14	17	13	30	26	13	0.0%	-0.71 [-1.50 , 0.09]	+	?	−	+	+	+
✓ Vong, 2011	5.6	4.5	38	7.6	6.4	38	28.9%	-0.36 [-0.81 , 0.10]	+	+	+	+	+	+
✓ Shimo, 2021	1.4	2.3	20	1.8	3.2	17	22.0%	-0.14 [-0.79 , 0.51]	+	+	?	+	+	+
✕ Amorin, 2019	5.7	5.3	34	6	5.7	31	0.0%	-0.05 [-0.54 , 0.43]	+	+	−	+	+	+
✕ Iles, 2011	74.6	27.6	489	71.6	27.3	410	0.0%	0.11 [-0.02 , 0.24]	+	+	−	+	+	+
✓ Rordigues, 2021	8.4	2.8	30	7.7	2.63	30	26.8%	0.25 [-0.25 , 0.76]	?	?	?	+	+	+
✕ Ben-Ami, 2017	6.7	6.4	94	4	6.2	95	0.0%	0.43 [0.14 , 0.72]	?	+	−	+	+	+
✓ Holden, 2024	26.7	16.9	21	15.8	14	19	22.3%	0.69 [0.04 , 1.33]	+	+	?	+	+	+
**Total (HKSJ[a])**			**109**			**104**	**100.0%**	**0.09 [-0.64 , 0.81]**						

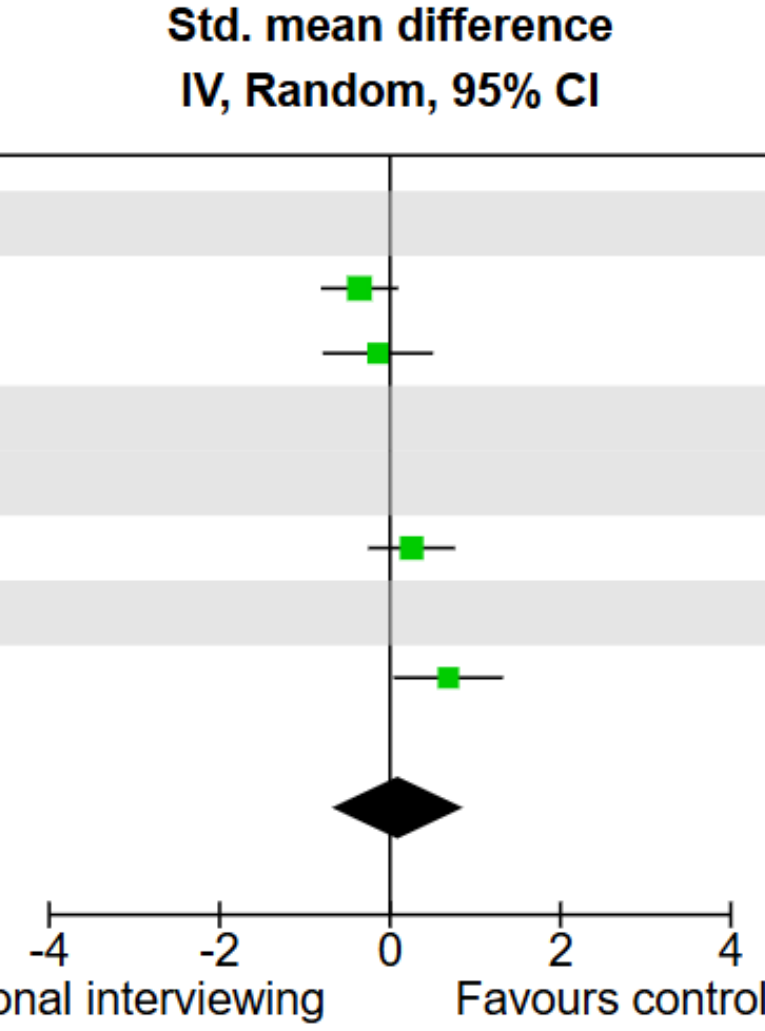

Test for overall effect: T = 0.38, df = 3 (P = 0.73)

Heterogeneity: Tau^2 (REML[b]) = 0.13; Chi^2 = 7.84, df = 3 (P = 0.05); I^2 = 61%

Footnotes

[a]CI calculated by Hartung-Knapp-Sidik-Jonkman method.

[b]Tau^2 calculated by Restricted Maximum-Likelihood method.

Risk of bias legend

(A) Random sequence generation (selection bias)
(B) Allocation concealment (selection bias)
(C) Blinding of participants and personnel (performance bias)
(D) Incomplete outcome data (attrition bias)
(E) Selective reporting (reporting bias)
(F) Other bias

Supplementary Material S13: Funnel plot for meta-analyses investigating motivational interviewing for change in physical activity

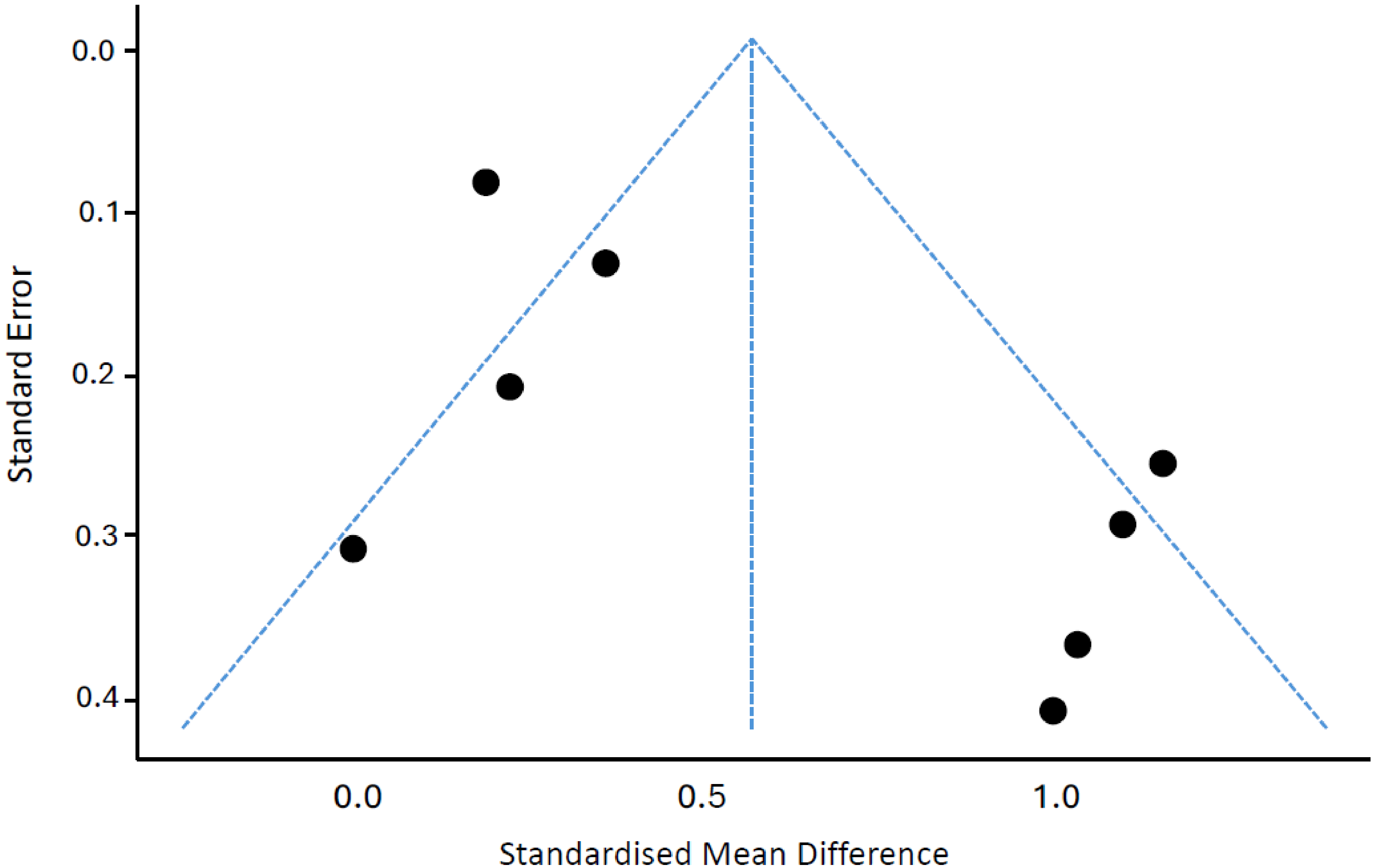

Supplementary Material S14: Funnel plot for meta-analyses investigating motivational interviewing for change in pain

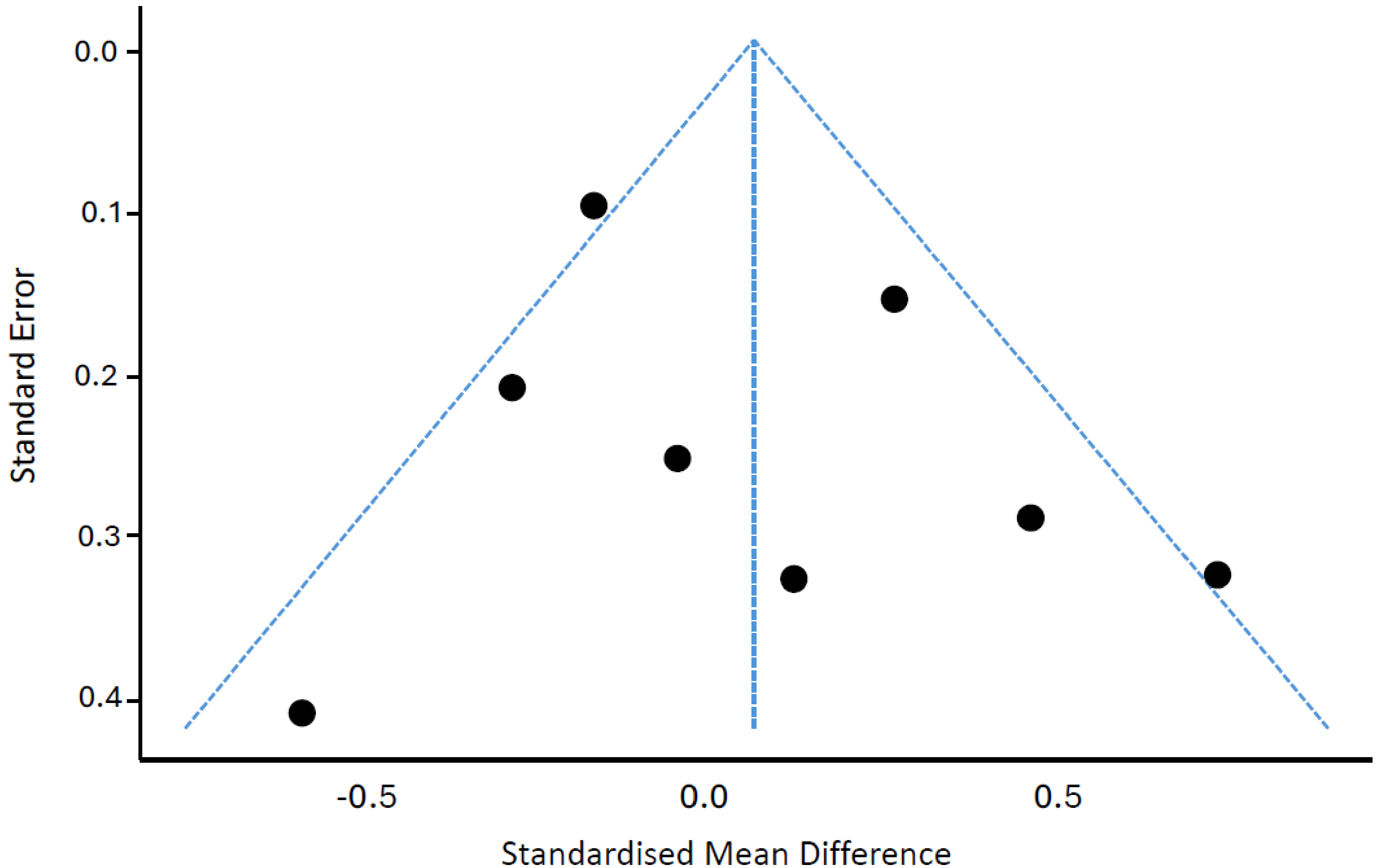

Supplementary Material S15: Funnel plot for meta-analyses investigating motivational interviewing for change in disability-related outcomes

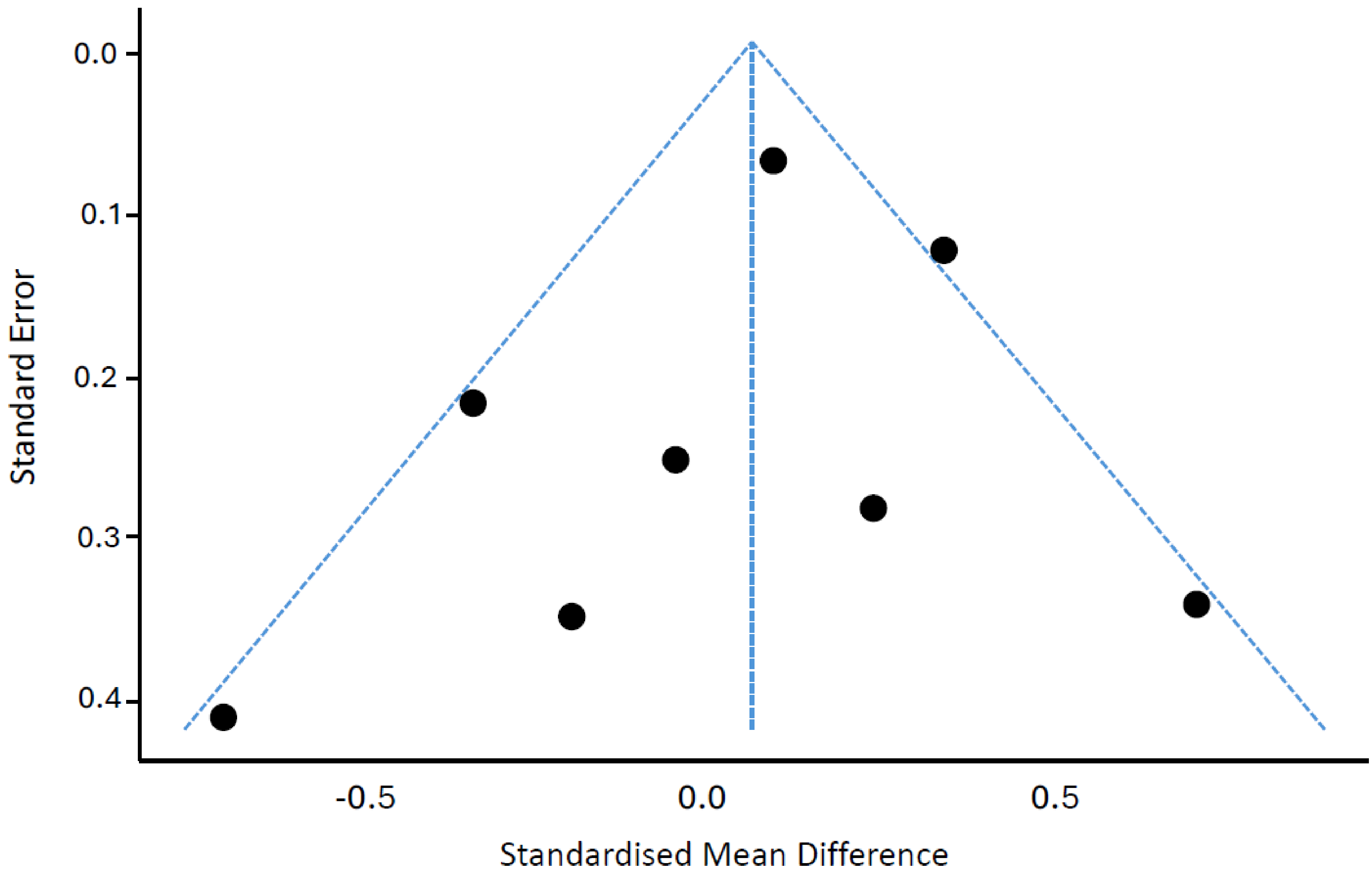

Supplement: Supplementary file 1 [file kaag040_supplementary_data.pdf]
